# Hazelnut Shell-Talc-Carbon Black Hybrid High-Density Polyethylene Biocomposites: Formulation-Property Relationships for Potential Rigid Food-Packaging Applications

**DOI:** 10.3390/polym18202546

**Published:** 2026-10-16

**Authors:** İdris Karagöz, Yeliz Karadeniz, Harun Sepetçioğlu, Volkan Daştan, Erdem Çınar

**Affiliations:** 1 Department of Polymer Materials Engineering, Faculty of Engineering, Yalova University, Yalova 77200, Türkiye; 2 Zamia Composite Research and Development Technology Joint Stock Company, ULUTEK Technopark, Nilüfer 16110, Türkiye; 3 Department of Chemical Engineering, Faculty of Engineering, Yalova University, Yalova 77200, Türkiye; yelizkaradenizw@gmail.com; 4 Department of Metallurgical and Materials Engineering, Faculty of Technology, Selçuk University, Konya 42075, Türkiye; harunsepet@selcuk.edu.tr; 5 Research and Development Department, Alterna Gıda Sanayi Pazarlama ve Tic. A.Ş., Akçaburgaz Mah. 592. Sok. No: 8/2, Esenyurt 34522, Türkiye; erdem.cinar@zuber.com

**Keywords:** high-density polyethylene, hazelnut shell, hybrid biocomposites, talc, melt processing, rigid food packaging

## Abstract

Agricultural residues offer opportunities to reduce virgin polymer consumption, although balancing mechanical performance, thermal stability, and processability in multicomponent composites remains challenging. This study developed high-density polyethylene (HDPE) hybrid biocomposites incorporating hazelnut-shell (HS) powder, talc, a carbonaceous additive, and maleic-anhydride-modified linear low-density polyethylene (LLDPE-g-MA) through sequential formulation. Mechanical, physical, processing, thermal, spectroscopic, and morphological properties were evaluated. The formulations exhibited distinct property trade-offs. The HS/talc formulation (PEHSCB4) achieved the highest flexural strength (38.87 MPa), 10.6% above neat HDPE, while maintaining comparable tensile strength. PEHSCB5 exhibited the highest tensile modulus (963.3 MPa) and Shore D hardness (71.8), whereas PEHSCB2 and PEHSCB3 achieved the highest V-notched impact strengths (~29 kJ/m^2^), compared with 12.78 kJ/m^2^ for neat HDPE. Melt flow rates ranged from 0.22 to 3.90 g/10 min, with all formulations remaining processable by extrusion and injection molding. Water absorption increased with HS incorporation but remained below 0.36% for all formulations after 240 h of immersion. Thermal analysis revealed formulation-dependent changes in crystallinity and initial degradation behavior. FTIR and SEM confirmed the polyethylene structure and heterogeneous multiphase morphologies. Overall, sequential formulation enabled property tailoring, supporting further evaluation for potential rigid food-packaging applications.

## 1. Introduction

Plastic materials remain indispensable in food-packaging applications because of their low density, cost-effectiveness, ease of processing, moisture resistance, chemical durability, and ability to provide the mechanical integrity required during handling, transportation, and storage. Nevertheless, the extensive consumption of short-lived packaging products, dependence on fossil-derived feedstocks, and accumulation of post-consumer plastic waste have intensified efforts to reduce the use of virgin petroleum-based polymers [1,2]. Among the strategies proposed for this purpose, the incorporation of agricultural and agro-industrial residues into thermoplastic matrices is particularly attractive because it can simultaneously create value-added utilization pathways for underused biomass and partially replace conventional polymer in the final product [1,2,3]. However, the environmental motivation for incorporating agricultural residues alone is insufficient to establish their suitability for packaging applications. The resulting materials must also maintain an appropriate balance of mechanical integrity, thermal stability, moisture resistance, processability, and dimensional performance [1,2,3]. For materials intended for direct food contact, regulatory requirements such as migration and sensory assessments constitute additional validation steps [1].

Recent studies have demonstrated the feasibility of incorporating lignocellulosic agricultural residues into polyethylene-based materials intended for food-packaging applications. Morcillo-Martín et al. incorporated cellulose fibers obtained from avocado pruning residues into bio-based polyethylene (BioPE) at concentrations of 10–40 wt.% and demonstrated that fiber loading and maleated-polyethylene compatibilization strongly affected mechanical performance [4]. The same research line subsequently showed that BioPE/avocado-fiber composites could be thermoformed into rigid food-packaging trays, with formulations containing up to 30 wt.% fiber being successfully converted into functional packaging structures [5]. More recently, tomato-pruning-derived cellulose fibers were incorporated into BioPE at concentrations of up to 40 wt.%, producing substantial changes not only in mechanical behavior but also in water absorption, heat-sealing performance, and migration [6]. Importantly, increasing lignocellulosic content increased water sensitivity and introduced additional constraints at high filler concentrations [6]. These findings demonstrate that replacing part of the polymer with agricultural residues is technically feasible, but also reveal a fundamental formulation challenge: increasing bio-based filler content does not necessarily improve all properties required for packaging simultaneously.

A major source of this challenge is the physicochemical incompatibility between lignocellulosic fillers and non-polar polyolefin matrices. Plant-derived fillers contain cellulose, hemicellulose, lignin, and hydroxyl-containing surface functionalities, whereas polyethylene is predominantly hydrophobic and non-polar. Insufficient filler–matrix interactions can therefore result in inefficient stress transfer, interfacial voids, filler pull-out, increased moisture sensitivity, and deterioration of selected mechanical properties [3,7]. Maleic-anhydride-grafted polyolefins are frequently employed to improve interfacial interactions in such systems because their functional groups can interact with lignocellulosic surfaces while the polyolefin backbone remains compatible with the matrix [4,8]. Nevertheless, compatibilizer addition should not be regarded as universally beneficial. Morcillo-Martín et al., for example, identified an optimum MAPE concentration in avocado-fiber/BioPE composites, beyond which the reinforcing efficiency decreased [4]. Consequently, compatibilizer concentration must be considered as an integral formulation variable rather than simply as an additive expected to continuously improve composite performance.

Hazelnut shell is a promising agro-industrial residue for particulate thermoplastic composites because it can be converted into a lignocellulosic filler through relatively straightforward drying, grinding, and particle-size control without requiring the isolation of purified cellulose fibers. Its incorporation into polyethylene matrices has already been demonstrated. Salasinska and Ryszkowska investigated polyethylene-waste composites containing ground hazelnut shell and reported substantial composition-dependent changes in dimensional stability, physical characteristics, mechanical performance, and thermal behavior [9]. Therefore, the scientific question is no longer whether hazelnut shell can be incorporated into polyethylene. Rather, a more relevant issue is how hazelnut-shell loading and its interaction with additional formulation components can be controlled to reduce the typical trade-offs associated with lignocellulosic particulate reinforcement.

These trade-offs arise because particulate fillers rarely improve all engineering properties simultaneously. Increasing the concentration of a relatively rigid filler can restrict polymer-chain mobility and increase stiffness, while ductility and impact resistance may decrease as a consequence of stress concentration, poor dispersion, or insufficient interfacial adhesion [7,10]. Furthermore, the performance of agricultural fillers is strongly dependent on their origin, morphology, particle size, composition, and interaction with the polymer matrix. Ogah et al. compared rice husk, corncob, walnut shell, and wood flour in recycled HDPE and demonstrated considerable differences in flexural and impact performance among the agro-waste fillers [10]. Accordingly, agricultural residues cannot be considered passive or interchangeable substitutes for polymer; their incorporation requires formulation strategies capable of balancing renewable filler content against the engineering requirements of the final application.

One approach to achieving such a balance is the use of hybrid filler systems in which lignocellulosic and inorganic constituents perform complementary functions. Previous studies have demonstrated the potential of this concept in polyolefin composites. Karagöz et al. investigated hazelnut shell and wollastonite in an elastomer-modified polypropylene matrix and showed that the mechanical and thermal performance of the resulting composites was strongly dependent on the hybrid formulation [11]. The hybridization concept was subsequently examined in HDPE systems containing walnut shell and nanoclay, where the simultaneous presence of lignocellulosic and inorganic fillers produced formulation-dependent changes in mechanical, thermal, and morphological properties [12]. More recently, Karagöz et al. investigated HDPE composites reinforced with walnut shell and nano-calcium carbonate and demonstrated that the relative contribution of the lignocellulosic and mineral phases significantly influenced the mechanical, thermal, crystallization, and morphological response of the hybrid composites [13]. Collectively, these studies indicate that combining agricultural residues with inorganic fillers can provide greater control over the overall property profile than relying on a single lignocellulosic filler alone.

Talc represents a particularly relevant mineral component for extending this hybridization strategy. Its platelet morphology and inherent rigidity can influence the stiffness, dimensional response, and crystallization behavior of polyolefin matrices. Carbon black, in contrast, is a carbonaceous particulate additive capable of modifying stiffness, hardness, crystallization behavior, and other functional characteristics of polyethylene. Parvin et al. investigated talc- and carbon-black-filled HDPE across a wide range of filler concentrations and demonstrated that both fillers substantially increased Young’s modulus, although their effects on tensile and flexural performance differed [14]. Importantly, talc and carbon black were evaluated as separate composite series rather than being incorporated together within the same formulation [14]. Their results therefore demonstrate both the reinforcing potential and the distinct structure–property responses associated with these fillers, while also suggesting that their simultaneous incorporation into a more complex hybrid system requires specific investigation rather than extrapolation from single-filler composites.

The stiffness–toughness trade-off associated with carbonaceous reinforcement has also been demonstrated in recent polyethylene systems. Kazeruni et al. investigated commercial and functionalized recycled carbon black in Bio-HDPE and reported substantial increases in tensile modulus and hardness with increasing carbon-black loading [15]. Carbon black also modified crystallization behavior and acted as a nucleating agent in the polyethylene matrix. However, these improvements in rigidity were accompanied by pronounced reductions in maximum strain and impact resistance at higher filler contents [15]. These findings reinforce an important principle for multi-component formulation design: an additive that improves one performance parameter may simultaneously compromise another. Carbon black concentration should therefore be evaluated within the complete composite formulation rather than being considered independently.

The same principle applies to mineral reinforcement and interfacial compatibilization. Talc can contribute to rigidity but may adversely affect strength when stress transfer and dispersion are insufficient [14], whereas maleated polyethylene can improve interactions between lignocellulosic particles and the HDPE matrix but does not necessarily produce monotonic improvements with increasing concentration [4,8]. A multi-component system containing hazelnut shell, talc, carbon black, and LLDPE-g-MA therefore presents a more complex formulation problem than conventional binary composites. Within such a system, hazelnut shell can serve as the agro-industrial lignocellulosic component and partial substitute for HDPE, talc as the mineral particulate reinforcement, carbon black as an additional functional particulate modifier, and LLDPE-g-MA as the interfacial compatibilizer. However, the individual contributions of these constituents cannot necessarily be extrapolated to multi-component formulations, because the resulting properties may depend on filler combinations, interfacial interactions, and changes in the polymer-rich phase. The practical challenge is to determine whether these chemically and morphologically distinct constituents can be combined while maintaining an acceptable balance among strength, stiffness, toughness, thermal behavior, and processing response.

Despite considerable progress in lignocellulosic/polyolefin composites, the available literature has predominantly addressed comparatively simpler material systems, including polyethylene/hazelnut shell [9], HDPE/agricultural particulate fillers [10], lignocellulosic–mineral hybrid polyolefin composites [11,12,13], separately prepared HDPE/talc and HDPE/carbon-black composites [14], and Bio-HDPE/carbon-black composites [15]. Although these investigations establish the relevance of individual agricultural, mineral, and carbonaceous fillers, their findings do not directly establish the property relationships of HDPE formulations incorporating these components within the same material system. In particular, the combined consideration of particulate hazelnut-shell residue, talc, carbon black, and maleated polyethylene remains insufficiently documented in the studies discussed above. A further gap concerns the comparative assessment of stiffness, strength, impact resistance, melt-flow behavior, and thermal characteristics across progressively developed single-filler, binary-filler, and multi-filler formulations. Addressing this issue is particularly relevant for rigid packaging applications, where maximizing either agricultural-residue loading or a single mechanical property is less meaningful than achieving a balanced overall performance profile.

Accordingly, the present study investigates HDPE-based hybrid biocomposites containing hazelnut-shell powder, talc, a carbonaceous additive, and LLDPE-g-MA through a sequential formulation-development strategy. Rather than employing a factorial optimization design, the formulations were structured to compare single-filler, binary-filler, and multi-filler systems and to examine the formulation response associated with effect of increasing the compatibilizer content within the multi-filler formulation. The principal contribution of this work is the systematic comparison of these formulation groups within a common experimental framework, with particular emphasis on identifying property trade-offs rather than assuming that increasing formulation complexity necessarily improves overall performance. The resulting materials were evaluated in terms of mechanical properties, density, melt-flow behavior, thermal characteristics, spectroscopic features, and fracture morphology to establish formulation–property relationships and assess the balance of performance relevant to potential rigid food-packaging applications. The study therefore provides a comparative basis for identifying formulations with different performance advantages while recognizing that the independent effects of individual constituents cannot be fully separated using the adopted sequential design. The suitability of the developed materials for direct food contact is not claimed in the present study and would require subsequent application-specific validation, including migration and sensory assessments.

## 2. Materials and Methods

### 2.1. Materials and Composite Formulations

High-density polyethylene (HDPE), commercially designated as InnoPlus HD8100M (PTT Global Chemical Public Company Limited, Bangkok, Thailand), was used as the polymer matrix. According to the manufacturer’s technical data, HD8100M has a density of 0.952 g cm^−3^ and a melt flow rate (MFR) of 0.25 g/10 min. Hazelnut shells obtained from the Tombul cultivar grown in the Giresun region of Türkiye were used as the lignocellulosic filler. The shells were dried, ground, and sieved to obtain a particle fraction between 75 and 100 µm, which was used throughout the composite preparation. This size range represents the selected sieve fraction rather than a particle size distribution determined by instrumental analysis. Talc, with a manufacturer-specified particle size below 44 µm and a density of 2.72 g cm^−3^, was supplied by Asil Kimya (Istanbul, Türkiye) and used as the mineral filler.

Carbon black (CB) was introduced into the formulations using Hostalen CRP 100 BLACK (LyondellBasell, Frankfurt am Main, Germany), a carbon-black-containing HDPE compound. Consequently, carbon black was not introduced as a separately weighed particulate powder. The amount of Hostalen CRP 100 BLACK added to each formulation was calculated based on its specified carbon black content to provide a target CB concentration of 0.5 wt.% in the final composite. The HDPE carrier fraction contributed by Hostalen CRP 100 BLACK was accounted for when determining the total polymer composition. Accordingly, Table 1 reports the actual amount of Hostalen CRP 100 BLACK added to the CB-containing formulations. Bondyram^®^ 4108 (Polyram Plastic Industries Ltd., Ram-On, Israel), a maleic-anhydride-modified linear low-density polyethylene (LLDPE-g-MA), was used as the compatibilizer. According to the manufacturer’s technical data, Bondyram^®^ 4108 has a density of 0.92 g cm^−3^, a melt flow index of 1.5 g/10 min at 190 °C/2.16 kg, and a melting point of 122 °C.

The formulations were developed using a sequential formulation-development strategy rather than a full factorial experimental design. LLDPE-g-MA was incorporated at 3 wt.% in the filler-containing formulations, except for PEHSCB7, in which its content was increased to 5 wt.% to examine the response of the multi-component system to a higher compatibilizer level. Because a filler-free HDPE/LLDPE-g-MA control was not included, the individual contribution of LLDPE-g-MA cannot be quantitatively separated from the effects associated with the fillers. Accordingly, comparisons among the formulations are interpreted in terms of overall formulation–property relationships rather than as independent effects of individual components.

The formulations were designed to progressively examine the behavior of single-filler, binary-filler, and multi-filler systems within the HDPE matrix. PEHSCB0, consisting of neat HDPE, was used as the reference material. PEHSCB1, PEHSCB2, and PEHSCB3 were formulated as single-filler systems containing 20 wt.% hazelnut shell, 5 wt.% talc, and 0.5 wt.% carbon black, respectively, at a constant LLDPE-g-MA content of 3 wt.%. PEHSCB4 and PEHSCB5 were binary-filler systems containing hazelnut shell/talc and hazelnut shell/carbon black, respectively. PEHSCB6 incorporated hazelnut shell, talc, and carbon black simultaneously at 20, 5, and 0.5 wt.%, respectively, while maintaining the LLDPE-g-MA content at 3 wt.%. PEHSCB7 contained the same filler combination as PEHSCB6, but the LLDPE-g-MA content was increased from 3 to 5 wt.%. This formulation strategy enabled the performance of the single-, binary-, and multi-filler systems to be compared while also allowing the influence of increased compatibilizer content within the multi-filler formulation to be assessed. The compositions of all formulations are presented in Table 1.

### 2.2. Preparation of Hazelnut-Shell Powder and Composite Specimens

Prior to grinding, the hazelnut shells were dried at 110 °C for 4 h. The dried shells were subsequently ground using a Demsan plant and spice grinder (Demsan, Istanbul, Türkiye; 4500 W, 25,000 rpm). The ground material was classified using a sieve shaker, and the particle fraction between 75 and 100 µm was collected and used for composite preparation.

The constituents of each formulation were weighed according to the compositions given in Table 1 and melt compounded using a GS-005 co-rotating twin-screw extruder (Nanjing GS-Mach Equipment Co., Ltd., Nanjing, China) equipped with 21 mm diameter screws and an L/D ratio of 36:1. The screw speed was maintained at 210 rpm, and the temperatures of the seven heating zones were set sequentially at 150, 160, 170, 180, 190, 200, and 210 °C, from the feeding section toward the die. Following melt compounding, the extruded strands were cooled and pelletized.

**Figure 1:**
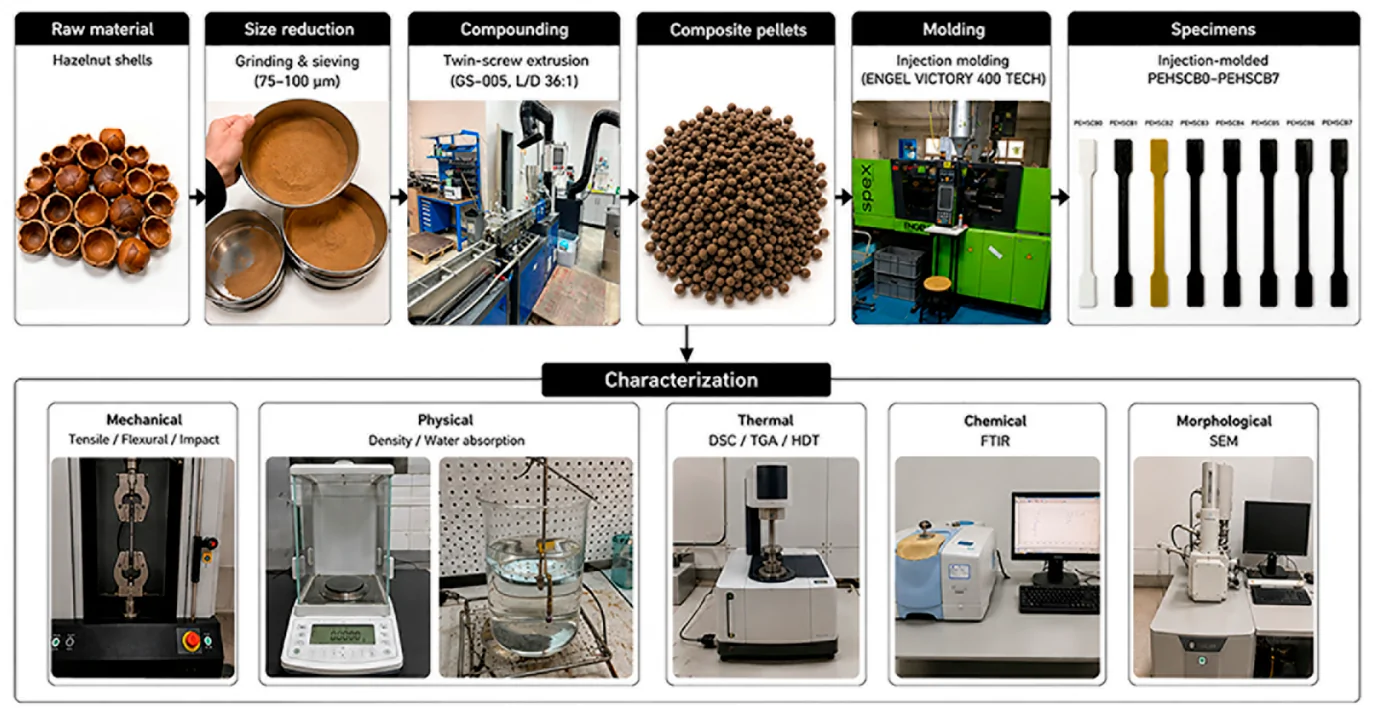
Schematic representation of the preparation and characterization workflow of the HDPE/hazelnut-shell hybrid biocomposites.

**Figure 2:**
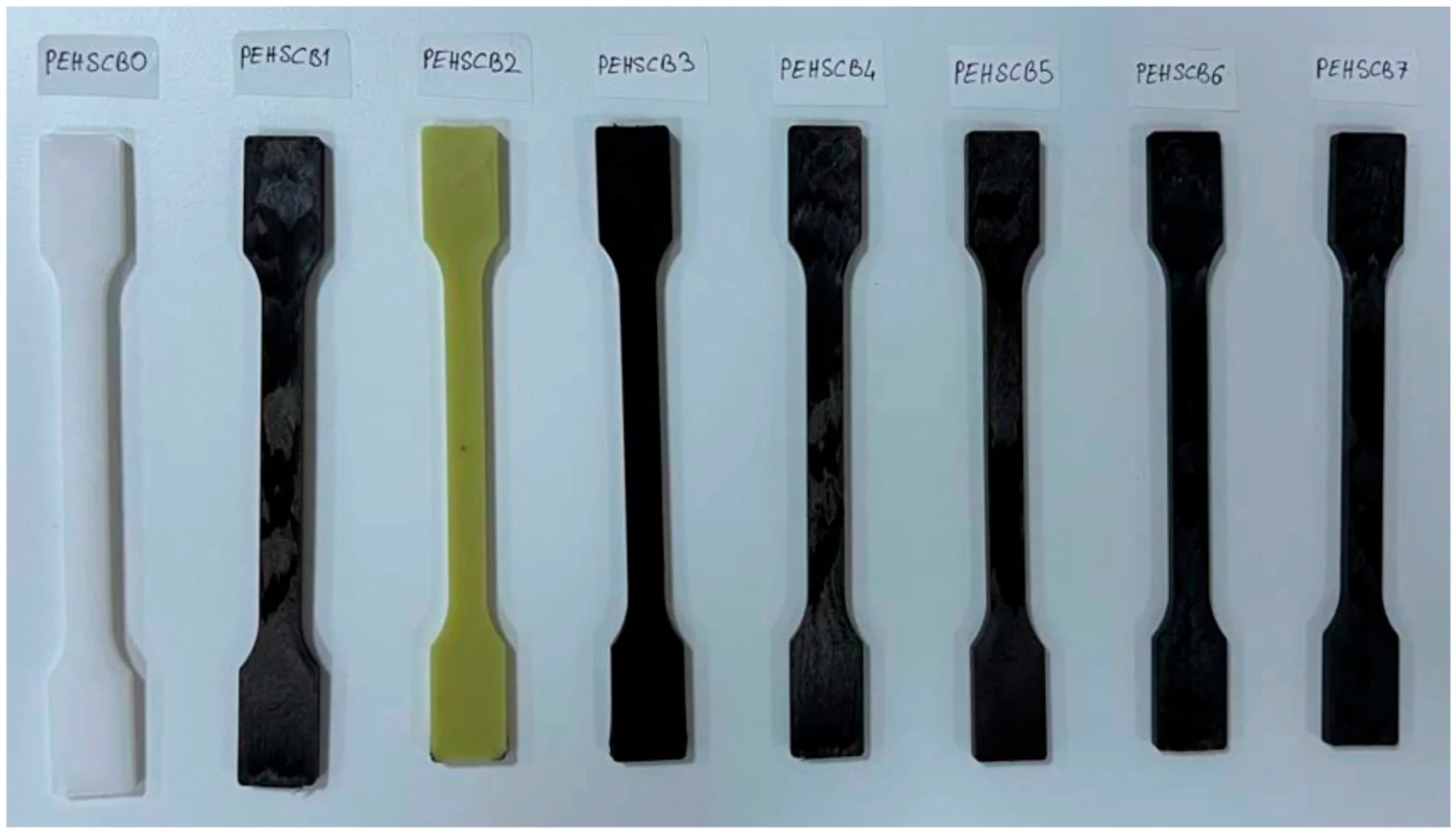
Representative injection-molded specimens of neat HDPE (PEHSCB0) and the investigated composite formulations (PEHSCB1–PEHSCB7).

Prior to injection molding, the resulting pellets were dried at 60 °C for 1 h. Standard test specimens were subsequently produced using an Engel Victory 400 Tech injection molding machine (ENGEL Austria GmbH, Schwertberg, Austria). The barrel/nozzle temperature profile was set sequentially at 170, 180, 190, 200, and 210 °C, from the rear barrel zone toward the nozzle. The mold temperature was maintained at 40 ± 5 °C. The injection pressure and injection speed were 80 bar and 50 mm s^−1^, respectively, while the holding pressure was 55 bar. The total molding cycle time was 35 s. All formulations listed in Table 1, including PEHSCB0 (neat HDPE), were processed using the same compounding and injection-molding conditions to facilitate direct comparison among the different filler combinations. All formulations were successfully compounded and injection molded under these conditions without observable processing difficulties. The overall preparation and characterization workflow is illustrated in Figure 1, while representative injection-molded specimens of the investigated formulations are shown in Figure 2.

### 2.3. Mechanical Characterization

Tensile tests were performed according to ISO 527-1 using a Zwick Z020 universal testing machine (ZwickRoell GmbH & Co. KG, Ulm, Germany) equipped with a 20 kN load cell. Strain was measured using an extensometer. The tensile modulus was determined at a crosshead speed of 1 mm/min, after which the test speed was increased to 50 mm/min for determination of the remaining tensile properties. Five specimens (*n* = 5) were tested for each formulation.

Three-point flexural tests were performed according to ISO 178 using standard specimens with dimensions of 80 × 10 × 4 mm, a support span of 64 mm, and a span-to-thickness ratio of 16:1. The crosshead speed was 1.7 mm/min, and five specimens (*n* = 5) were tested for each formulation.

Both V-notched and unnotched Charpy impact tests were performed using an Instron 120D impact testing machine (Instron, Norwood, MA, USA) equipped with a 7.5 J pendulum, in accordance with ISO 179. Rectangular specimens measuring 80 × 10 × 4 mm were used, with V-notches prepared according to the relevant ISO 179 requirements. Ten specimens were tested for each formulation, and the mean impact strength was determined.

Shore D hardness measurements were performed using a Loyka M01D analog durometer (Loyka, Istanbul, Türkiye) in accordance with ISO 868. Five independently prepared specimens were tested for each formulation, with one representative measurement obtained from each specimen (*n* = 5).

### 2.4. Physical Characterization

Density was determined according to ISO 1183 using the Archimedes method with a RADWAG AS 220.R2 PLUS balance (RADWAG, Radom, Poland) equipped with a density determination kit. Isopropyl alcohol (ρ = 0.785 g/cm^3^) was used as the immersion liquid.

The melt flow rate (MFR) of neat HDPE and the composite formulations was determined in accordance with ISO 1133 at 190 °C under a load of 5 kg using an MF20 melt flow index tester (Instron CEAST, Turin, Italy). The MFR values were expressed in g/10 min.

Water absorption was evaluated following ASTM D570 using rectangular specimens measuring 30 × 20 × 4 mm. Before immersion, the specimens were conditioned at room temperature and their initial dry masses (W_0_) were recorded using an analytical balance (Sartorius Cubis II, Germany). The specimens were then fully immersed in distilled water in a borosilicate glass vessel (IKA, Germany) under static conditions. At predetermined intervals of 1, 4, 8, 24, 48, 72, 96, 120, 144, 168, 192, 216, and 240 h, the specimens were removed from the water, gently wiped with absorbent paper to eliminate surface moisture, and immediately weighed to determine their masses after immersion (*W_t_*). Water absorption (*WA*) was calculated using Equation (1):

(1) $$\mathrm{WA}\;\left(\%\right)=\frac{W_{t}-W_{0}}{W_{0}}\;\times \;100$$

where *W*_0_ is the initial dry mass and *W_t_* is the mass measured after immersion for a specified period. Three independent specimens were tested for each formulation, and the results were reported as mean ± standard deviation.

### 2.5. Thermal, Spectroscopic, and Morphological Characterization

Differential scanning calorimetry (DSC) was performed using a Mettler Toledo TGA/DSC2 STARe system (Mettler Toledo, Greifensee, Switzerland). Approximately 10 mg of each sample was analyzed under a nitrogen atmosphere at a flow rate of 50 mL/min. The samples were first heated from 25 to 300 °C at a heating rate of 10 °C/min, subsequently cooled from 300 °C to 25 °C at 10 °C/min, and then reheated to 300 °C at 10 °C/min. The melting temperature (*T_m_*) and melting enthalpy (Δ*H_m_*) were determined from the second-heating curves to minimize the influence of the previous thermal history.

The degree of crystallinity of the polyethylene phase (*X_C_*) was calculated according to Equation (2):

(2) $$X_{C}(\%)=\frac{{∆H}_{m}}{{\;∆H}_{m}^{O}\;\times {\;\omega }_{\mathrm{PE}}}\times \;100$$

where Δ*H_m_* is the melting enthalpy obtained from the second-heating DSC curve, ${\;∆H}_{m}^{O}$ is the heat of fusion of 100% crystalline polyethylene (293 J/g), and *ω_PE_* is the total polyethylene-based mass fraction in the formulation, including the directly incorporated HDPE, LLDPE-g-MA, and the HDPE carrier present in the carbon-black-containing compound. The carbon black itself, hazelnut-shell powder, and talc were excluded from this fraction. For the calculation, LLDPE-g-MA was included in the polyethylene-based phase.

Thermogravimetric analysis (TGA) was performed using a Seiko Exstar TG/DTA 6300 instrument (Seiko Instruments, Chiba, Japan). Approximately 10 mg of each sample was heated from room temperature to 600 °C at a heating rate of 10 °C min^−1^ under a nitrogen flow of 50 mL min^−1^. The resulting thermogravimetric curves were used to evaluate the thermal degradation behavior and thermal stability of the formulations.

Fourier-transform infrared (FTIR) spectroscopy was performed using a PerkinElmer Spectrum 100 FTIR spectrometer (PerkinElmer, Waltham, MA, USA) over the wavenumber range of 4000–400 cm^−1^. The spectra were used to identify characteristic functional groups and evaluate changes in the spectral features of the composite formulations.

The fracture surfaces of impact-tested specimens were examined using a Zeiss EVO LS10 scanning electron microscope (Zeiss, Oberkochen, Germany) to investigate filler dispersion, particle–matrix interfacial characteristics, particle pull-out, void formation, and fracture morphology. Prior to SEM examination, the specimens were sputter-coated with an approximately 100 Å gold layer to provide electrical conductivity. Micrographs were acquired at an accelerating voltage of 20 kV.

### 2.6. Statistical Analysis

Mechanical test results were expressed as mean ± standard deviation. Statistical differences among the formulations were evaluated using one-way analysis of variance (ANOVA), followed by Tukey’s honestly significant difference (HSD) post-hoc test for multiple comparisons. Tensile and flexural properties and V-notched Charpy impact strength were analyzed across all formulations. For unnotched Charpy impact strength, specimens exhibiting no fracture (“No break”) were excluded from the statistical analysis. Differences were considered statistically significant at *p* < 0.05. Different superscript letters were used to indicate statistically significant differences among the formulations within each property.

## 3. Results

### 3.1. Mechanical Properties

**Figure 3:**
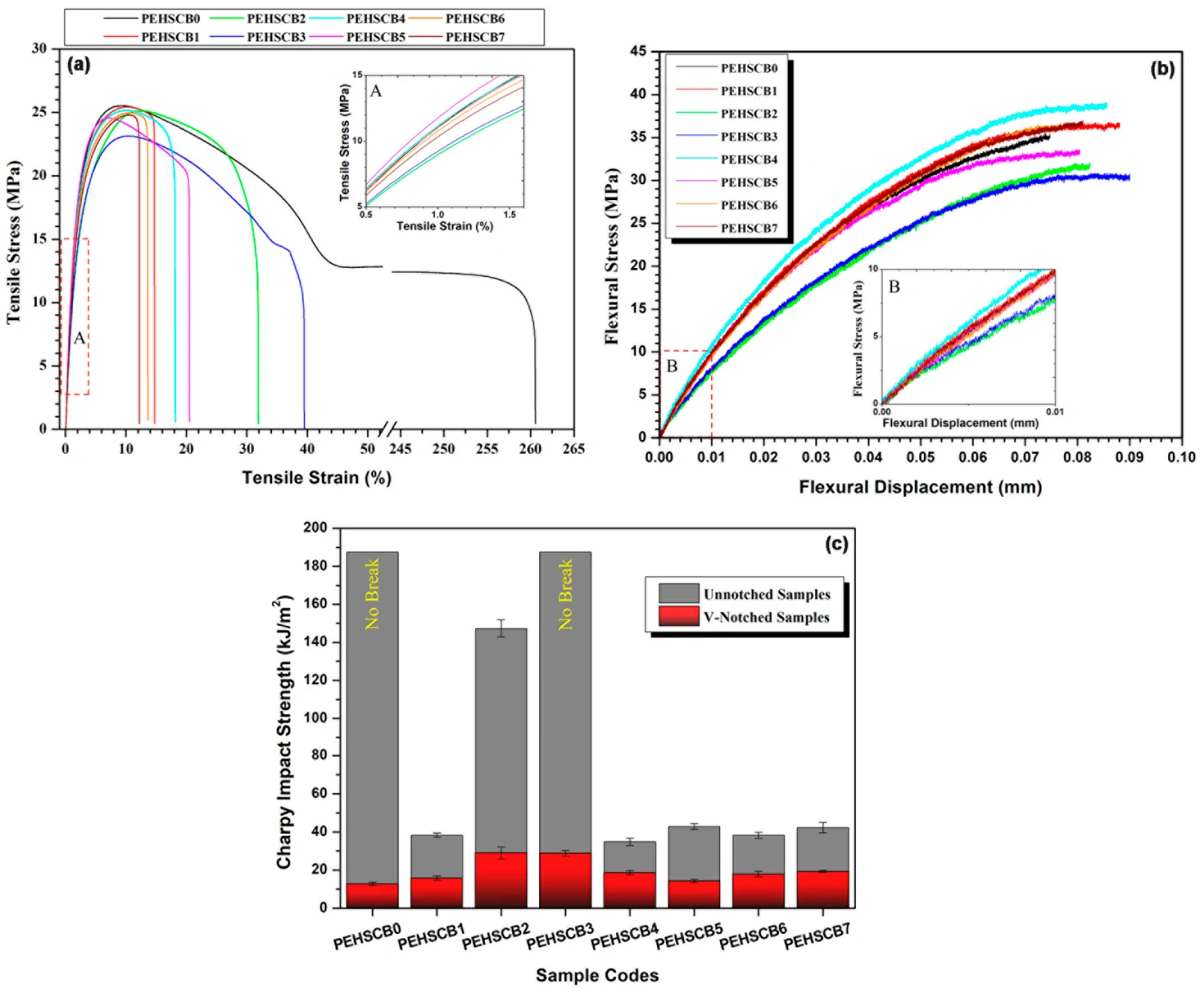
Mechanical behavior of neat HDPE and the developed composite formulations: (**a**) tensile stress–strain curves, (**b**) flexural stress–displacement curves, and (**c**) V-notched and unnotched Charpy impact strength.

The tensile and flexural properties of neat HDPE and the investigated composites are summarized in Table 2, while their corresponding stress–strain curves are presented in Figure 3a and Figure 3b, respectively. The results reveal a pronounced formulation-dependent mechanical response, with the different filler combinations affecting stiffness, strength, and deformation behavior to different extents. Overall, the compositional changes did not produce a uniform improvement or deterioration in mechanical performance; instead, clear trade-offs were observed among tensile stiffness, ductility, flexural resistance, and impact behavior.

The tensile stress–strain curves (Figure 3a) clearly show the transition from the highly ductile response of neat HDPE to the more restricted deformation of the composite formulations. The most pronounced change occurred in elongation at break, which decreased from 271.88% for neat HDPE to 12.80–45.22% for the composites. The reduction was particularly evident in the HS-containing formulations. This behavior can primarily be associated with the presence of rigid lignocellulosic particles, which restrict large-scale deformation of the polyethylene matrix and introduce heterogeneous regions at which stresses can become concentrated. Salasinska and Ryszkowska similarly attributed the pronounced reduction in elongation of hazelnut-shell-filled polyethylene to limitations imposed by the filler on polymer deformation [9]. Demirer et al. also observed a substantial decrease in elongation with increasing ground hazelnut-shell content in PP, accompanied by increased stiffness [16]. Such stiffness–ductility trade-offs are widely reported for nutshell-filled thermoplastics and depend strongly on filler loading, particle morphology, dispersion, and interfacial characteristics [17].

Despite this pronounced loss of ductility, the incorporation of 20 wt.% HS in PEHSCB1 caused only limited changes in tensile modulus and strength relative to neat HDPE. This is an important distinction because it indicates that the substantial reduction in deformation capacity was not accompanied by a comparable loss of tensile load-bearing capability. Similar concentration-dependent behavior has been reported for hazelnut-shell/HDPE composites, where moderate shell contents maintained or even slightly increased tensile strength, whereas higher filler contents resulted in deterioration as agglomeration, insufficient wetting, and interfacial voids became more prominent [9]. Therefore, the tensile response of PEHSCB1 suggests that HS incorporation primarily altered the deformation mechanism of the matrix rather than severely compromising its tensile strength.

PEHSCB2 and PEHSCB3 exhibited lower tensile moduli than neat HDPE despite containing talc and carbon black, respectively. Although rigid particulate fillers are often expected to increase stiffness, this behavior should not be interpreted solely in terms of the intrinsic rigidity of the fillers. Both formulations also contained 3 wt.% LLDPE-g-MA and consequently a reduced HDPE fraction, while the final mechanical response additionally depends on particle dispersion, matrix–particle interaction, and the microstructure developed during processing. The results therefore emphasize that the contribution of a filler cannot be considered independently of the complete formulation.

The binary HS-containing formulations further illustrate this point. PEHSCB5 exhibited the highest tensile modulus among the investigated materials, whereas PEHSCB4 retained tensile strength and modulus relatively close to those of neat HDPE. However, the ranking changed substantially under flexural loading (Figure 3b), where PEHSCB4 exhibited the highest overall performance, with flexural modulus and strength approximately 7.8% and 10.6% higher than neat HDPE, respectively. Thus, the HS/talc combination was particularly favorable under bending rather than tensile loading. Nevertheless, these improvements were accompanied by a substantial reduction in tensile ductility, as the elongation at break of PEHSCB4 decreased from 271.9% for neat HDPE to 15.1%. Its unnotched impact strength was also lower than that of neat HDPE, which did not fracture under the applied test conditions. Conversely, PEHSCB4 exhibited a higher V-notched impact strength (18.70 kJ/m^2^) than neat HDPE (12.78 kJ/m^2^), although it remained below the values obtained for PEHSCB2 and PEHSCB3. These results demonstrate that the improvement in flexural performance was accompanied by reduced deformation capacity and unnotched impact resistance, highlighting the need to consider stiffness and toughness together rather than evaluating either property independently.

This response is consistent with the general concept of hybrid lignocellulosic/mineral reinforcement, in which the mechanical behavior is determined by the combined effects of fillers with different stiffness, morphology, and interfacial characteristics rather than simply by total filler content. In our previous work on hazelnut-shell/wollastonite-filled PP composites, the introduction of a secondary mineral reinforcement substantially modified the mechanical response of the lignocellulosic-filled polyolefin system [11]. Likewise, our previous study on walnut-shell/nano-CaCO_3_-reinforced HDPE demonstrated that combining a shell-derived lignocellulosic phase with a mineral phase can alter the balance among stiffness, strength, and deformation behavior [13]. These earlier findings support the present observation that the performance of PEHSCB4 is better interpreted as the response of the combined HS/talc formulation rather than as an isolated talc effect.

A comparable stiffness–ductility trade-off was also observed in our walnut-shell/nanoclay-reinforced HDPE composites, where the simultaneous presence of lignocellulosic and inorganic phases modified matrix deformation and the overall mechanical response [12]. Together with the present results, these findings indicate that hybridization does not necessarily maximize every mechanical property simultaneously; instead, its principal advantage may lie in tailoring the balance between stiffness, strength, and deformation according to the intended application. From this perspective, PEHSCB4 represents a favorable compromise among the investigated formulations for potential rigid packaging applications. It combined the highest flexural modulus (1018.5 MPa) and flexural strength (38.9 MPa) with a tensile strength (25.6 MPa) close to that of neat HDPE (26.0 MPa), while maintaining a V-notched impact strength approximately 46% higher than that of the unfilled matrix. Although PEHSCB5 exhibited a higher tensile modulus and PEHSCB2 and PEHSCB3 provided greater notched impact resistance, these advantages were not accompanied by similarly favorable flexural performance. Therefore, PEHSCB4 offers a comparatively balanced mechanical property profile for applications in which resistance to bending and retention of tensile strength are important, provided that its reduced ductility and unnotched impact resistance are acceptable for the intended service conditions.

The effect of increasing compatibilizer content was evaluated directly by comparing PEHSCB6 and PEHSCB7, which contained the same filler combination but 3 and 5 wt.% LLDPE-g-MA, respectively. Increasing the compatibilizer content did not improve tensile stiffness or strength and produced little change in elongation at break. Under flexural loading, however, the mean flexural strength increased while the modulus remained essentially comparable. Maleated polyethylene is generally used in lignocellulosic/polyethylene composites to promote interaction between the non-polar polyolefin matrix and polar lignocellulosic surfaces, thereby facilitating stress transfer across the interface. Nevertheless, the effectiveness of compatibilization is concentration dependent and does not necessarily increase monotonically with compatibilizer loading [17,18]. Accordingly, the PEHSCB6–PEHSCB7 comparison suggests that increasing LLDPE-g-MA from 3 to 5 wt.% shifted the balance of mechanical properties rather than producing a general reinforcement effect.

The Charpy impact results (Figure 3c and Table 3) provide further evidence that tensile ductility and resistance to rapid crack propagation did not follow the same trend. In the unnotched condition, neat HDPE and PEHSCB3 did not fracture under the applied test conditions, whereas substantial differences were observed among the remaining formulations. The relatively large scatter observed for PEHSCB1 should also be noted when interpreting its unnotched impact response.

The V-notched results provide a more direct comparison across all formulations. The most notable response was obtained for PEHSCB2 and PEHSCB3, whose impact strengths were approximately 2.3 times that of neat HDPE. This enhancement is particularly interesting because both formulations exhibited substantially lower elongation at break than the unfilled matrix. The result demonstrates that tensile ductility alone is not sufficient to predict notched impact resistance. This difference can be explained by the distinct loading and deformation conditions of the two tests. Tensile elongation reflects the ability of a specimen to undergo deformation under relatively slow loading, whereas V-notched impact strength reflects its resistance to fracture under rapid loading and localized stress concentration. Consequently, reduced tensile ductility does not necessarily imply lower resistance to notched impact fracture. Although both formulations contained 3 wt.% LLDPE-g-MA, their enhanced impact performance cannot be attributed solely to a toughening effect associated with the compatibilizer, since the same concentration was used in the other filler-containing formulations. Moreover, PEHSCB2 and PEHSCB3 differed from neat HDPE in both filler composition and polymer composition, preventing the independent contributions of the fillers and compatibilizer from being determined. Furthermore, the absence of hazelnut-shell particles in PEHSCB2 and PEHSCB3 may have reduced lignocellulosic particle-associated stress concentrations compared with the corresponding hybrid formulations. Under rapid crack propagation, energy can be dissipated through several local mechanisms, potentially including matrix deformation around dispersed particles, crack deflection, interfacial debonding, particle pull-out, and microvoid formation. Crack deflection may increase the effective crack propagation path, whereas localized particle–matrix debonding and microvoid formation may promote energy dissipation through associated matrix deformation. Particle pull-out may also contribute under suitable interfacial conditions, although its occurrence depends on particle characteristics and fracture morphology. The possible contribution of localized matrix deformation to the observed impact response is further considered in the morphological analysis presented in Section 3.5. However, the mechanical results and available morphological observations do not independently establish the occurrence or relative contributions of these individual fracture mechanisms. Therefore, crack deflection, particle–matrix debonding, and particle pull-out are considered possible energy-dissipation mechanisms rather than experimentally verified processes based on the available evidence.

The literature likewise shows that the influence of lignocellulosic particles on impact resistance is strongly formulation dependent. Decreases in impact strength have frequently been reported as nutshell loading increases because rigid particles restrict matrix deformation and facilitate crack initiation [16,19]. In contrast, other hazelnut-shell/polyolefin systems have exhibited impact resistance above that of the neat polymer at selected compositions [17]. These apparently contrasting observations indicate that particle concentration, morphology, dispersion, interfacial adhesion, matrix characteristics, and the presence of secondary modifiers collectively determine impact behavior. Consequently, the high notched impact resistance of PEHSCB2 and PEHSCB3 should be regarded as a formulation-specific response, rather than being generalized as an intrinsic effect of talc or carbon black.

The impact response of the multi-filler formulations provides additional evidence of this compositional dependence. Increasing LLDPE-g-MA from 3 wt.% in PEHSCB6 to 5 wt.% in PEHSCB7 produced a modest increase in V-notched impact resistance despite the absence of a corresponding improvement in tensile properties. This behavior reinforces the conclusion that compatibilizer concentration affects the balance between stiffness, deformation, and fracture resistance rather than uniformly enhancing all mechanical properties.

Shore D hardness measurements further demonstrated the formulation-dependent mechanical response of the composites (Table 4). Neat HDPE (PEHSCB0) exhibited a hardness of 65.8 ± 0.4, whereas the incorporation of 20 wt.% hazelnut shell in PEHSCB1 increased the hardness to 70.0 ± 0.7. A similar increase in hardness following nutshell incorporation has previously been reported for both PP- and HDPE-based composites [13,16]. The highest hardness values were obtained for PEHSCB5 (71.8 ± 1.1) and PEHSCB3 (71.6 ± 1.1), corresponding to increases of approximately 9.1% and 8.8%, respectively, compared with neat HDPE. In contrast, PEHSCB2 showed only a slight increase to 66.2 ± 0.8, while PEHSCB4, PEHSCB6, and PEHSCB7 exhibited hardness values of 68.0 ± 1.6, 67.0 ± 0.7, and 67.6 ± 1.1, respectively. One-way ANOVA confirmed that the formulation had a statistically significant effect on Shore D hardness (*p* < 0.001). These results indicate that surface hardness was not directly proportional to the total particulate content but rather depended on the specific combination of formulation components and their resulting resistance to localized indentation. Interestingly, a similar formulation-dependent response was reported for walnut-shell/nano-CaCO_3_-filled HDPE, where walnut-shell incorporation increased Shore D hardness, whereas subsequent addition of the inorganic filler did not produce a further increase [13]. This observation is also consistent with the broader mechanical response of the present composites, as the formulations exhibiting the highest Shore D hardness did not necessarily exhibit the highest tensile or flexural modulus. Therefore, Shore D hardness should be regarded as a complementary indicator of localized surface resistance rather than a direct measure of the overall stiffness of the composites.

Taken together, the mechanical results demonstrate that no single formulation maximized all measured properties. PEHSCB5 provided the highest tensile stiffness, PEHSCB4 showed the most favorable flexural response, whereas PEHSCB2 and PEHSCB3 exhibited the greatest V-notched impact resistance. Increasing formulation complexity also did not automatically produce superior performance, as illustrated by the multi-filler PEHSCB6 and PEHSCB7 systems. Instead, the results demonstrate that filler type and combination, together with compatibilizer concentration, determine the final balance among stiffness, strength, ductility, and impact resistance.

From an application perspective, this balance is more informative than maximizing an individual mechanical property. For potential rigid packaging applications, retention of adequate tensile strength together with flexural resistance and resistance to handling-induced impact may be more relevant than preserving the exceptionally high elongation of neat HDPE. Among the investigated systems, PEHSCB4 is particularly noteworthy because the incorporation of 20 wt.% HS together with 5 wt.% talc produced the highest flexural performance while maintaining tensile properties relatively close to those of the neat matrix. Nevertheless, mechanical performance alone is insufficient to establish application suitability. The physical, thermal, and morphological results presented in the following sections are therefore considered together with the mechanical behavior to identify the formulations providing the most balanced overall performance.

To place the mechanical performance of the developed composites in the context of previous research, a comparison with selected HDPE-based natural-filler composites is presented in Table 5. Previous studies have reported considerable variations in mechanical properties depending on filler type, concentration, and compatibilization strategy [20,21]. In particular, Karagöz et al. [13] reported tensile strengths of 25.6 and 22.7 MPa for HDPE composites containing 20 wt.% walnut shell without and with 3 wt.% nano-CaCO_3_, respectively. The corresponding tensile strengths of PEHSCB1 and PEHSCB4 in the present study were 25.5 and 25.6 MPa, indicating comparable tensile performance despite differences in filler composition. However, the tensile moduli of PEHSCB1 (915.2 MPa) and PEHSCB4 (915.0 MPa) were higher than those reported for the corresponding walnut-shell-based composites (595.4 and 596.2 MPa, respectively), whereas their flexural strengths were lower. These findings highlight the formulation-dependent balance between stiffness and strength in HDPE-based biocomposites.

### 3.2. Thermal Properties

**Figure 4:**
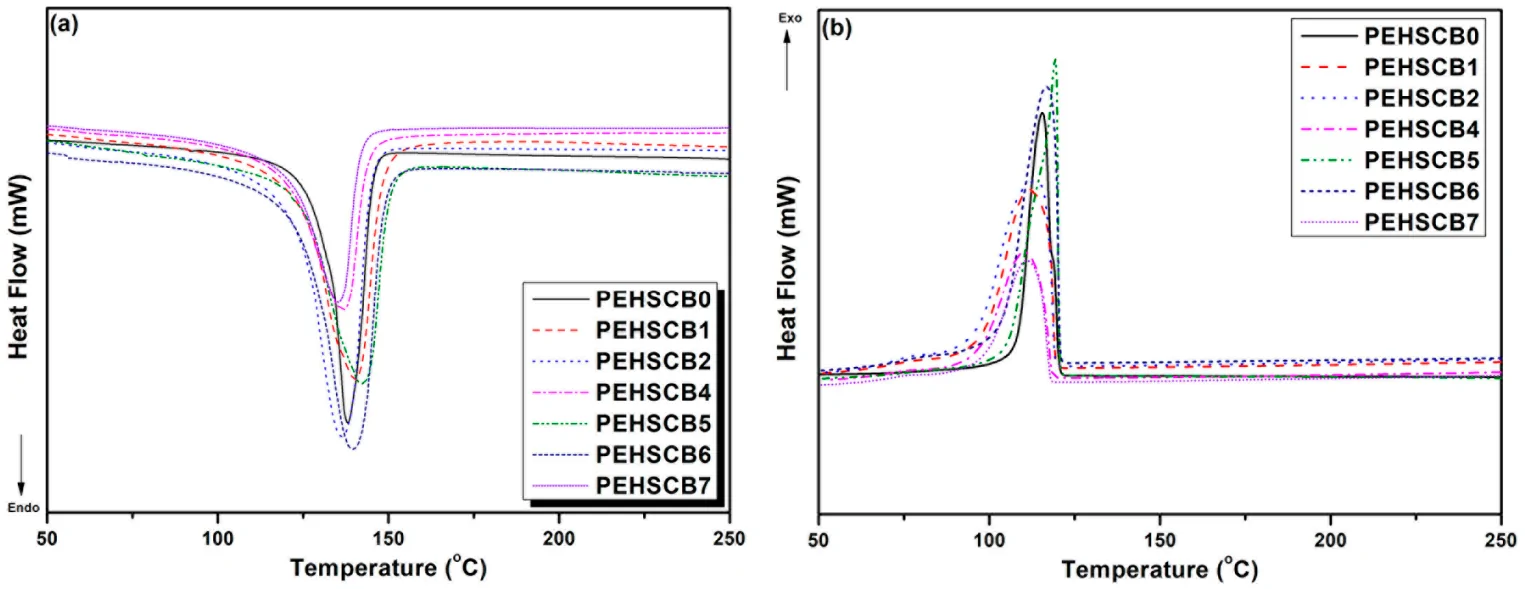
(**a**) Second-heating DSC thermograms and (**b**) cooling DSC thermograms of neat HDPE and the selected composite formulations.

The second-heating DSC thermograms of neat HDPE and the selected composite formulations are compared in Figure 4a, while the corresponding cooling thermograms are presented in Figure 4b. The melting enthalpy (ΔH_m_), melting temperature (T_m_), crystallization enthalpy (ΔHc), crystallization peak temperature (T_c_), and polyethylene-phase-normalized degree of crystallinity (Xc) values are summarized in Table 6. The degree of crystallinity was calculated by normalizing the measured melting enthalpy to the total polyethylene-based mass fraction of each formulation, including the directly incorporated HDPE, LLDPE-g-MA, and the HDPE carrier of the carbon-black-containing compound, using the heat of fusion of 100% crystalline polyethylene (293 J/g) as the reference value [22]. Neat HDPE (PEHSCB0) exhibited a T_m_ of 138.83 °C and an Xc of 67.08%. Overall, the formulations showed moderate variations in T_m_, ranging from 135.67 to 142.83 °C, whereas considerably greater differences were observed in Xc, which varied from 60.36 to 81.37%. Thus, the compositional changes affected the crystalline organization of the polyethylene phase more strongly than the position of the melting transition.

The cooling thermograms (Figure 4b) revealed formulation-dependent differences in crystallization behavior. The crystallization peak temperature ranged from 109.67 °C for PEHSCB4 to 118.17 °C for PEHSCB5, compared with 114.50 °C for neat HDPE. The crystallization enthalpy also varied among the formulations, decreasing from 219.95 J/g for neat HDPE to 147.81–188.78 J/g for the composites. However, these enthalpy values are expressed per unit mass of the entire specimen and are therefore influenced by differences in polymer content. Taken together, the cooling results indicate that the incorporated constituents modified the crystallization behavior of HDPE, although they do not independently establish a dominant crystallization mechanism.

The incorporation of hazelnut shell alone (PEHSCB1) slightly increased *T_m_* from 138.83 to 140.83 °C, while *X_c_* remained essentially unchanged at 67.21% compared with 67.08% for neat HDPE. This result indicates that the incorporation of 20 wt.% hazelnut shell did not substantially alter the final crystallinity of the polyethylene phase under the applied processing conditions. However, PEHSCB1 exhibited a lower Tc (111.17 °C) than neat HDPE (114.50 °C), suggesting that the hazelnut-shell-containing formulation shifted the crystallization peak toward lower temperatures without substantially changing the final crystalline fraction. In contrast, PEHSCB2 exhibited the lowest *X_c_* among the investigated formulations (60.36%), with a *T_m_* of 137.00 °C. Its Tc was also lower than that of neat HDPE (112.33 versus 114.50 °C), indicating that the presence of talc did not increase the crystallization peak temperature under the investigated conditions. Talc is known to promote heterogeneous nucleation in semicrystalline polymers and can modify the crystallization behavior of HDPE. However, its effect depends on factors including particle size, concentration, dispersion, and matrix characteristics [23,24]. Therefore, enhanced heterogeneous nucleation does not necessarily result in a proportional increase in the final crystalline fraction. Sánchez-Soto et al. [23] reported that talc increased the onset and peak crystallization temperatures of HDPE-based systems, while the effect on the crystalline fraction was particularly dependent on talc particle size. Similarly, Karrad et al. [24] demonstrated that fine talc modified the crystallization behavior of HDPE and that the crystalline fraction generally increased with talc content.

For PEHSCB4, containing both hazelnut shell and talc, *T_m_* and *X_c_* were 137.17 °C and 66.14%, respectively. The crystallinity was therefore comparable to that of neat HDPE and PEHSCB1, indicating that the simultaneous presence of hazelnut shell and talc did not produce an additive increase in the final crystalline fraction. The cooling results further showed that PEHSCB4 exhibited the lowest Tc (109.67 °C) among the investigated formulations, together with a crystallization enthalpy of 153.88 J/g. This shift toward lower crystallization temperatures suggests that the combined hazelnut-shell/talc formulation altered the crystallization process differently from the individual filler-containing formulations. This behavior is consistent with the complex nature of crystallization in particulate-filled HDPE, where heterogeneous nucleation, polymer-chain mobility, particle dispersion, and interfacial interactions can simultaneously affect crystal development. The dependence of crystallization behavior on filler characteristics and concentration has also been reported for HDPE/talc systems [23,24].

A more pronounced response was observed for PEHSCB5 and PEHSCB6. PEHSCB5 exhibited an *X_c_* of 76.60% and the highest *T_m_* of 142.83 °C, while PEHSCB6 showed the highest *X_c_* among the investigated formulations, reaching 81.37%, with a *T_m_* of 140.33 °C. Both formulations exhibited higher crystallization peak temperatures than neat HDPE, with Tc values of 118.17 °C for PEHSCB5 and 115.67 °C for PEHSCB6. Their crystallization enthalpies were 182.79 and 188.78 J/g, respectively. The higher Tc values indicate that crystallization occurred at higher temperatures during cooling, although the differences in crystallization enthalpy should be interpreted in relation to the polymer content of each formulation. The higher polyethylene-phase-normalized crystallinity of these formulations suggests that their combined formulation environment favored crystalline organization of the polyethylene phase. The presence of particulate fillers may provide heterogeneous nucleation sites, while filler characteristics, dispersion, and polymer–filler interactions can influence chain organization and the resulting crystallization behavior [23,24]. Nevertheless, because several formulation components are present simultaneously, the increase in *X_c_* cannot be attributed exclusively to a single filler or interfacial mechanism on the basis of DSC results alone. Furthermore, the influence of LLDPE-g-MA on crystallization should be considered composition-dependent, since previous studies have shown that maleated LLDPE can modify polyethylene crystallinity and crystalline organization rather than producing a universally increasing or decreasing effect [25,26].

A particularly relevant comparison can be made between PEHSCB6 and PEHSCB7, which contained similar proportions of HS, talc, and the carbon-black-containing component but differed in LLDPE-g-MA content. Increasing the compatibilizer content from 3 to 5 wt.% was accompanied by a decrease in *X_c_* from 81.37% to 65.11%, together with a reduction in *T_m_* from 140.33 to 135.67 °C. The cooling results also showed a decrease in Tc from 115.67 to 110.33 °C, accompanied by a reduction in crystallization enthalpy from 188.78 to 147.81 J/g. These observations provide additional evidence that the compositional change between PEHSCB6 and PEHSCB7 influenced the crystallization behavior of the polyethylene phase. This behavior suggests that the compositional adjustment associated with the higher LLDPE-g-MA content influenced the crystalline organization of the polyethylene phase. Previous studies have similarly demonstrated that the incorporation of LLDPE-g-MA can modify the crystallization behavior and crystalline organization of polyethylene-based systems [25,26]. Santos et al. [25] reported changes in crystallinity and crystallite characteristics in polyethylene-based blends containing LLDPE-g-MA, whereas Passador et al. [26] showed that LLDPE-g-MA exhibits lower crystallinity than HDPE-g-MA and can influence the crystalline/amorphous organization of polyethylene-based systems. Therefore, the decrease observed for PEHSCB7 should not be interpreted solely as a direct inhibitory effect of the compatibilizer on crystallization, but rather as a formulation-dependent modification of chain organization and crystal development.

The contribution of polyethylene crystallinity to the mechanical behavior of the composites must be considered alongside the reinforcing effects of the incorporated fillers. PEHSCB2, which exhibited the lowest crystallinity (60.36%), also showed a substantially lower tensile modulus (743.5 MPa) than neat HDPE (942.9 MPa). Although this observation is consistent with a possible contribution of reduced crystallinity to the lower stiffness, the remaining formulations did not exhibit a corresponding trend. PEHSCB6 showed the highest crystallinity (81.37%) but a lower tensile modulus (875.7 MPa) than neat HDPE, whereas PEHSCB5 exhibited the highest tensile modulus (963.3 MPa) at a lower crystallinity (76.60%). Furthermore, PEHSCB4 achieved the highest flexural strength (38.87 MPa) despite having a crystallinity (66.14%) comparable to that of neat HDPE (67.08%). A similar distinction was observed between PEHSCB6 and PEHSCB7, where the substantial reduction in crystallinity from 81.37% to 65.11% was accompanied by a slight increase in flexural strength from approximately 36.0 to 37.5 MPa. Collectively, these results indicate that the variations in polyethylene-phase crystallinity do not translate directly into corresponding changes in mechanical performance. The mechanical response of these multiphase composites is therefore governed by the combined contributions of crystalline organization, filler characteristics, particle distribution, interfacial interactions, and deformation behavior rather than crystallinity alone.

**Figure 5:**
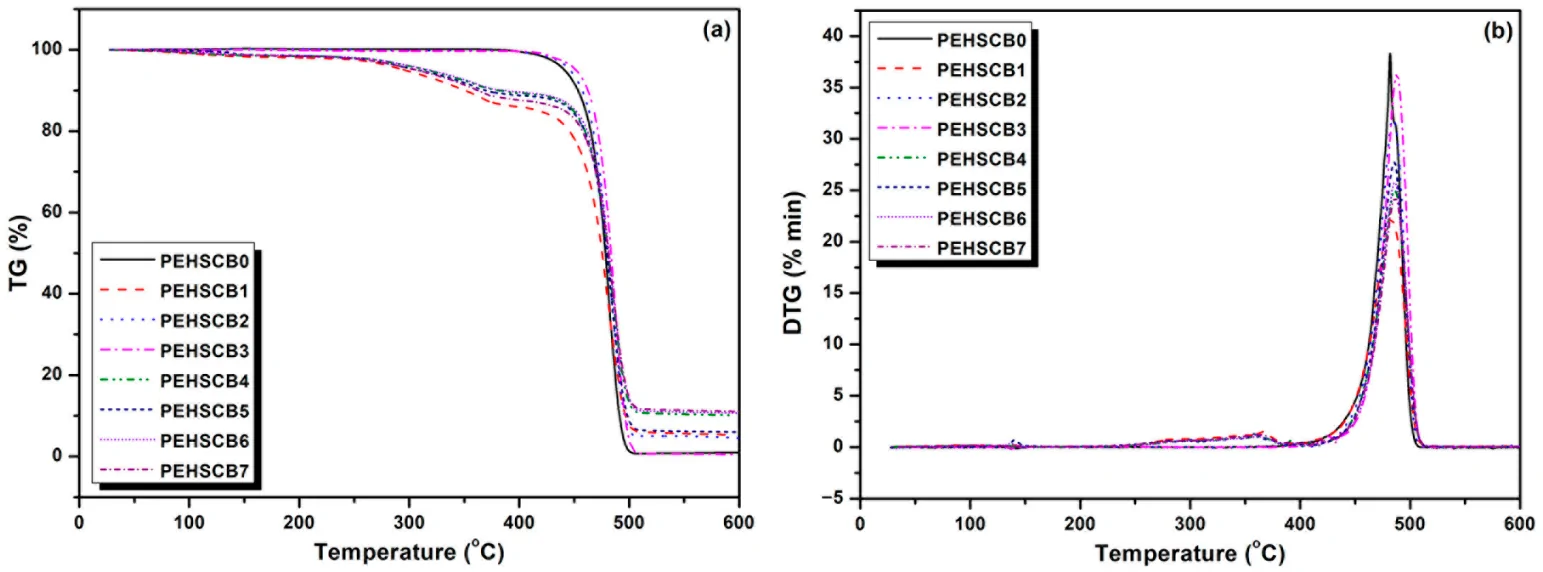
Thermal degradation behavior of neat HDPE and composite formulations: (**a**) TG curves and (**b**) DTG curves.

The thermal degradation behavior of neat HDPE and the composite formulations was evaluated by thermogravimetric (TG) and derivative thermogravimetric (DTG) analyses. The TG and DTG curves are presented in Figure 5a and Figure 5b, respectively, while the characteristic temperatures corresponding to 5% and 50% mass loss (T5 and T50), the temperature of maximum mass-loss rate (Tmax), and the residual mass at 600 °C are summarized in Table 7. Neat HDPE (PEHSCB0) exhibited predominantly single-stage thermal degradation, with T5, T50, and Tmax values of 457.4, 482.0, and 501.0 °C, respectively. In contrast, the hazelnut-shell-containing formulations showed an additional lower-temperature degradation region before the principal decomposition of the polyethylene matrix (Figure 5a). This behavior is characteristic of lignocellulosic-filled polyolefin composites and is associated with the degradation of lignocellulosic constituents at temperatures lower than those required for polyethylene decomposition [9,12,13]. Thermogravimetric analysis of hazelnut shell itself has also demonstrated multistage degradation associated with its lignocellulosic constituents, supporting the occurrence of an earlier mass-loss region when hazelnut shell is incorporated into the polymer matrix [27].

The influence of hazelnut shell was particularly evident in the T5 values. Incorporation of 20 wt.% hazelnut shell in PEHSCB1 decreased T5 from 457.4 to 365.8 °C, while PEHSCB5–PEHSCB7 exhibited similarly reduced values of 358.3–361.4 °C. The decrease in initial thermal stability is consistent with the presence of thermally less stable lignocellulosic constituents in the shell. Previous studies on nutshell-filled polyethylene systems have similarly reported earlier degradation associated with hemicellulose-, cellulose-, and lignin-containing fractions [9,12,13]. This interpretation is further supported by the thermal degradation behavior reported for hazelnut-shell biomass [27]. Interestingly, PEHSCB4 exhibited a substantially higher T5 of 426.8 °C than the other hazelnut-shell-containing hybrid formulations. This result indicates that the initial degradation response depended on the overall formulation rather than solely on the presence of the lignocellulosic filler. However, because several formulation components vary across the sample series, the comparatively high T5 of PEHSCB4 cannot be attributed exclusively to a single component. The comparatively high T5 of PEHSCB4 may nevertheless be considered consistent with the contribution of talc to the thermal response of HDPE-based systems. Previous studies have shown that talc-containing HDPE formulations can retain or increase the initial degradation temperature relative to the corresponding unfilled polymer, although the magnitude of this effect depends strongly on formulation and test conditions [28,29].

Despite the pronounced differences in T5, the T50 values remained within a relatively narrow range of 481.1–488.0 °C. PEHSCB1 showed the lowest T50 (481.1 °C), close to that of neat HDPE (482.0 °C), whereas PEHSCB3 exhibited the highest value (488.0 °C). The hybrid formulations PEHSCB4–PEHSCB7 showed closely grouped T50 values of 485.8–486.4 °C. Thus, although the lignocellulosic fraction promoted earlier mass loss in several formulations, it did not cause a comparable reduction in the temperature required to reach 50% overall mass loss. This distinction indicates that T5 is particularly sensitive to the lower-temperature degradation of the lignocellulosic fraction, whereas T50 more closely reflects the dominant high-temperature degradation of the polyethylene-rich phase.

A similar trend was observed for Tmax, which varied only from 501.0 to 506.3 °C. The highest Tmax was obtained for PEHSCB6 (506.3 °C), compared with 501.0 °C for neat HDPE. As evident from the DTG curves (Figure 5b), all formulations retained a dominant high-temperature degradation peak within a relatively narrow temperature interval. Therefore, incorporation of the particulate components affected the initial degradation behavior more strongly than the maximum-rate degradation event of the polyethylene-rich phase. Similar formulation-dependent responses have been reported for HDPE composites containing lignocellulosic shell fillers and inorganic particles [12,13]. Talc-filled HDPE systems have likewise been reported to retain a dominant polymer-degradation region despite changes in filler content [29,30]. Accordingly, the reduction in T5 observed for several hazelnut-shell-containing formulations should not be interpreted as an equivalent reduction in thermal stability throughout the entire degradation process.

The residual mass at 600 °C also exhibited a clear formulation dependence. Neat HDPE and PEHSCB3 left only 1.0% and 0.4% residue, respectively, whereas PEHSCB1 and the talc-containing PEHSCB2 showed residual masses of 5.1% and 4.2%, respectively. Considerably higher residues were observed for PEHSCB4 (9.9%), PEHSCB6 (10.5%), and PEHSCB7 (10.9%), all of which contained both hazelnut shell and talc. These higher residual fractions are consistent with the combined contributions of carbonaceous residue derived from the lignocellulosic component and the non-volatile mineral fraction. This interpretation is directly supported by Huang et al. [29], who observed increasing residual mass with increasing talc content in HDPE-based wood–plastic composites and attributed a substantial proportion of the remaining mass to the inorganic, non-volatile talc fraction. In their study, for example, the residual mass increased to 11.97% for an HDPE shell containing 10 wt.% talc, whereas unfilled HDPE left only 0.83% residue. Similar increases in final residue have been reported for hybrid HDPE composites containing lignocellulosic and inorganic particulate fillers [12,13]. Therefore, the higher residual mass of the hybrid formulations should not, by itself, be interpreted as evidence of improved thermal stability.

The combined presence of talc and a carbonaceous component in HDPE systems has also been examined by Mehrjerdi et al. [30], who studied HDPE containing carbon black together with talc contents of up to 35 wt.%. Their results further demonstrate that the thermal response of multi-component HDPE formulations depends on the combined formulation rather than simply on the behavior of an individual particulate component.

Comparison of PEHSCB6 and PEHSCB7 further showed that increasing the LLDPE-g-MA content from 3 to 5 wt.% resulted in only minor changes in T5 (361.4 to 359.7 °C), T50 (486.3 to 486.4 °C), Tmax (506.3 to 504.2 °C), and residual mass (10.5% to 10.9%). These relatively small differences suggest that increasing the compatibilizer content within this range did not substantially alter the overall thermal degradation behavior of the hybrid system. Nevertheless, because the change in compatibilizer content also modifies the relative polymer composition, this comparison is more appropriately interpreted as a formulation-dependent response rather than evidence of a specific compatibilization mechanism.

Overall, the TG/DTG results reveal two distinct aspects of the thermal behavior of the developed composites. Hazelnut-shell incorporation primarily affected the initial degradation region, as reflected by the reduction in T5, whereas T50 and Tmax remained comparatively close to those of neat HDPE. The principal high-temperature degradation process therefore remained predominantly associated with the polyethylene-rich phase. In parallel, the hybrid formulations containing hazelnut shell and talc exhibited higher residual masses due to the combined contributions of lignocellulosic-derived carbonaceous residue and the non-volatile mineral fraction. Consequently, the thermal stability of these multi-component composites cannot be adequately described by a single degradation parameter. From a processing perspective, the lower-temperature degradation associated with the lignocellulosic fraction should therefore be considered when evaluating the thermal behavior of the developed materials under melt-processing conditions. Nevertheless, even the lowest T5 value measured in the present study (358.3 °C for PEHSCB5) remained substantially above the maximum melt-processing temperature of 210 °C used during composite preparation. This demonstrates a substantial difference between the applied processing temperature and the temperature corresponding to 5% mass loss under the investigated TGA conditions. However, this comparison should be interpreted cautiously because dynamic TGA under nitrogen does not reproduce the residence time, shear, and possible oxidative conditions encountered during melt processing.

### 3.3. Physical and Processing Properties

The density and melt flow rate (MFR) values of neat HDPE and the composite formulations are presented in Table 8. The density of neat HDPE (PEHSCB0) was 0.95 g/cm^3^, while the composite formulations exhibited values ranging from 0.95 to 1.04 g/cm^3^. The incorporation of 20 wt.% hazelnut shell in PEHSCB1 increased the density to 1.01 g/cm^3^, corresponding to an increase of approximately 6.1% relative to neat HDPE. PEHSCB2, containing 5 wt.% talc, exhibited a density of 0.99 g/cm^3^, whereas PEHSCB3 showed a density of 0.95 g/cm^3^, remaining essentially comparable to that of PEHSCB0. Among the hybrid formulations, PEHSCB4, PEHSCB6, and PEHSCB7 exhibited the highest density of 1.04 g/cm^3^, while PEHSCB5 showed a density of 1.01 g/cm^3^.

The higher densities observed for PEHSCB4, PEHSCB6, and PEHSCB7 are consistent with the presence of talc in combination with the other formulation components. Talc has a substantially higher density than the polyethylene matrix; accordingly, incorporation of talc into HDPE is generally associated with an increase in composite density. Mehrjerdi et al. similarly reported an increase in the specific density of HDPE composites with increasing talc content [30]. The effect of talc on the density of polyethylene systems has also been demonstrated under molten conditions, where increasing talc concentration resulted in an approximately linear increase in melt density [31]. Nevertheless, the present results indicate that the incorporation of the particulate components produced only a moderate change in the overall density of the materials. Even the highest measured density of 1.04 g/cm^3^ represents an increase of only approximately 9.2% relative to neat HDPE. Thus, the hybrid formulations retained densities close to that of the polymer matrix despite the incorporation of hazelnut shell, talc, and the carbonaceous component. From the perspective of potential rigid packaging applications, this relatively limited increase is relevant because improvements or changes in mechanical and thermal performance should be considered together with the mass-related consequences of filler incorporation.

In contrast to the relatively narrow variation in density, the MFR showed a pronounced formulation-dependent response, ranging from 0.22 to 3.90 g/10 min at 190 °C under a 5 kg load. Neat HDPE (PEHSCB0) exhibited an MFR of 0.22 g/10 min. The incorporation of 20 wt.% hazelnut shell in PEHSCB1 increased the MFR to 1.50 g/10 min, whereas PEHSCB2 and PEHSCB3 exhibited values of 1.00 and 2.63 g/10 min, respectively. Among the hybrid formulations, PEHSCB4 exhibited an MFR of 0.65 g/10 min, while PEHSCB5 showed the highest value of 3.90 g/10 min. In contrast, PEHSCB6 and PEHSCB7 both exhibited an MFR of 0.25 g/10 min, approaching the value measured for neat HDPE.

The broad range of MFR values indicates that melt-flow behavior was considerably more sensitive to formulation changes than density. Under identical testing conditions, higher MFR values generally indicate lower apparent resistance to melt flow, suggesting potentially more favorable flow characteristics during melt processing. Melt-flow characteristics of particulate-filled polyethylene systems depend not only on the total filler content but also on filler characteristics, polymer–filler interactions, dispersion, polymer composition, and the resulting melt structure. Rheological studies on talc-filled HDPE have demonstrated that the melt response is dependent on both material composition and testing conditions, and that particulate incorporation can modify the viscoelastic behavior of the polymer melt [31]. Importantly, the relationship between filler incorporation and MFR is not necessarily universal. For HDPE/hazelnut-shell composites, Taşdemir reported a decrease in MFI with increasing hazelnut-shell concentration [32]. In the present study, however, PEHSCB1 exhibited a higher MFR than neat HDPE. This difference indicates that the melt-flow response of the present system cannot be attributed solely to the presence of hazelnut shell, particularly because the formulations also contain LLDPE-g-MA and, depending on the formulation, talc and a carbonaceous component.

This interpretation is further supported by the substantial differences observed among formulations containing the same hazelnut-shell concentration. PEHSCB1, PEHSCB4, PEHSCB5, PEHSCB6, and PEHSCB7 all contained 20 wt.% hazelnut shell, yet their MFR values ranged from 0.25 to 3.90 g/10 min. In particular, PEHSCB5 exhibited the highest MFR (3.90 g/10 min), whereas PEHSCB6 and PEHSCB7 exhibited substantially lower and identical values of 0.25 g/10 min. Therefore, the results point to a strong dependence of melt flow on the specific combination of formulation components rather than on hazelnut-shell content alone. Moreover, increasing the LLDPE-g-MA content from 3 wt.% in PEHSCB6 to 5 wt.% in PEHSCB7 did not produce a measurable change in MFR under the applied test conditions, as both formulations exhibited the same value. This suggests that, within these two formulations, the increase in compatibilizer content did not translate into a detectable change in melt flow.

The comparatively high MFR of PEHSCB5 should also be interpreted cautiously. Although its value was substantially higher than those of both PEHSCB1 and the other hazelnut-shell-containing hybrid formulations, MFR represents a standardized empirical measure of melt flow under a specified temperature and load rather than a complete rheological characterization of the material. Consequently, the observed differences should not be directly interpreted as equivalent changes in zero-shear viscosity or used alone to establish a specific molecular or interfacial mechanism. Furthermore, MFR measurements alone cannot fully characterize the shear-rate- and temperature-dependent rheological behavior of polymer melts under injection-molding conditions, where melt flow is also influenced by processing parameters and mold geometry. Detailed rheological measurements would be required to resolve the individual contributions of the polymer phase, filler–matrix interactions, and particulate network formation to the observed flow behavior [31].

Despite the broad variation in MFR, all formulations were successfully melt-compounded by twin-screw extrusion and subsequently processed by injection molding under the conditions employed in this study. Therefore, even the low-MFR PEHSCB6 and PEHSCB7 formulations remained processable within the experimental processing window. This demonstrates that the lower MFR values did not preclude melt processing under the investigated conditions, although MFR alone is insufficient to predict flow behavior under the shear conditions encountered during extrusion and injection molding. From a processing perspective, the relatively higher MFR values of PEHSCB3 and PEHSCB5 suggest lower resistance to melt flow under the standardized test conditions, which may be advantageous for filling geometrically complex or relatively thin-walled injection-molded components. Conversely, formulations exhibiting lower MFR values, particularly PEHSCB6 and PEHSCB7, may require greater consideration of melt temperature, injection pressure, and flow-path geometry to achieve adequate mold filling. Nevertheless, successful specimen preparation under the applied injection-molding conditions indicates that these formulations can be processed without requiring a high MFR. Taken together, the density and MFR results demonstrate that the sequential changes in formulation produced relatively modest variations in solid-state density but substantially greater differences in melt-flow behavior. These findings reinforce the importance of evaluating processing characteristics alongside mechanical and thermal performance when assessing the overall property balance of the developed hybrid HDPE biocomposites. For potential rigid packaging applications, the selection of an appropriate formulation should therefore consider not only melt-flow characteristics but also the required mechanical performance, component geometry, and processing conditions. Although the present results demonstrate the feasibility of producing injection-molded specimens, additional rheological characterization and application-specific molding trials are necessary to assess mold-filling behavior, dimensional stability, and process robustness under industrial manufacturing conditions.

**Figure 6:**
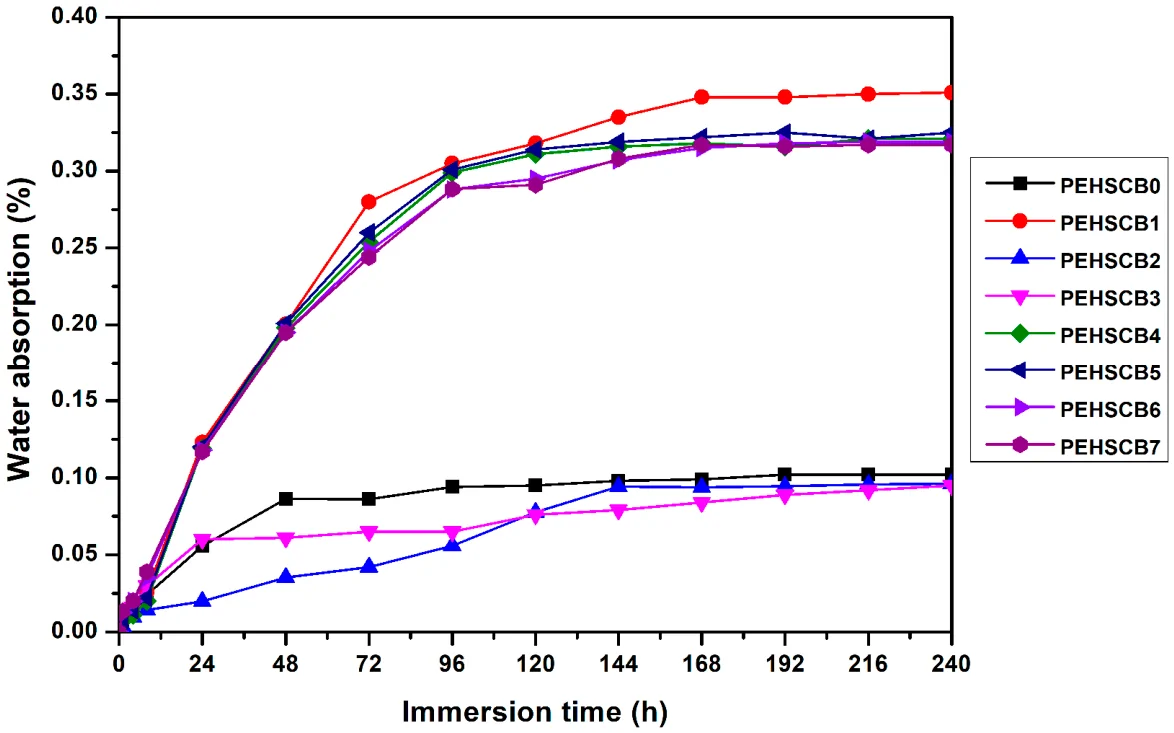
Water absorption behavior of neat HDPE and composite formulations during immersion in distilled water for 240 h.

Figure 6 presents the water absorption behavior of neat HDPE and the composite formulations during 240 h of immersion in distilled water. All formulations exhibited time-dependent water uptake, with the absorption rate gradually decreasing and approaching an apparent plateau after approximately 168–192 h. However, the magnitude of water absorption varied depending on the filler composition. The formulations without hazelnut shell powder (HS), namely PEHSCB0, PEHSCB2, and PEHSCB3, exhibited relatively low water absorption, reaching 0.102%, 0.096%, and 0.095%, respectively, after 240 h. These results indicate that the individual incorporation of talc or carbon black did not increase water uptake compared with neat HDPE under the investigated conditions, consistent with the predominantly hydrophobic nature of the polyethylene matrix.

In contrast, the incorporation of 20 wt.% HS substantially increased water absorption, with PEHSCB1 reaching the highest final value of 0.351%, approximately 3.44 times that of neat HDPE. This increase can primarily be attributed to the hydrophilic nature of the lignocellulosic constituents of HS, particularly cellulose and hemicellulose, whose hydroxyl groups facilitate interactions with water molecules. Additionally, interfacial imperfections and microvoids between the filler particles and the polymer matrix may provide pathways for moisture penetration. Similar behavior has been reported in natural-filler-reinforced polyethylene composites, where water absorption is influenced by filler characteristics, particle size, and polymer–filler interfacial interactions [33,34,35]. Tufan and Ayrilmis [36] also investigated hazelnut husk-reinforced recycled HDPE composites, providing relevant context for the utilization of hazelnut-derived lignocellulosic residues in polyethylene-based materials. However, hazelnut husk differs from the hard hazelnut shell used in the present study.

The HS-containing hybrid formulations exhibited slightly lower final water absorption than PEHSCB1, with values of 0.321%, 0.325%, 0.319%, and 0.317% for PEHSCB4, PEHSCB5, PEHSCB6, and PEHSCB7, respectively. These values correspond to reductions of approximately 7–10% relative to PEHSCB1, suggesting that the incorporation of additional fillers may influence moisture transport within the composite structure. However, the relatively small differences among the hybrid formulations do not establish a systematic reduction in water absorption attributable to either talc or carbon black. Furthermore, increasing the LLDPE-g-MA content from 3 wt.% (PEHSCB6) to 5 wt.% (PEHSCB7) resulted in only a marginal difference in final water absorption (0.319% versus 0.317%). Previous studies have reported that maleic-anhydride-based treatments and compatibilization can improve polymer–lignocellulosic filler interactions and reduce moisture uptake [33,35,37]. Nevertheless, the present results indicate that increasing the compatibilizer concentration did not produce a pronounced additional improvement under the investigated conditions.

Overall, the results demonstrate that HS incorporation is the primary factor governing water absorption in the investigated HDPE biocomposites, whereas the additional fillers exert comparatively limited effects. Despite the increased moisture uptake associated with HS, all formulations exhibited final water absorption values below 0.36% after 240 h. These findings provide preliminary information regarding the moisture resistance of the developed materials for potential rigid packaging applications. Nevertheless, further application-specific testing is required to establish their long-term dimensional stability and suitability for direct food contact.

### 3.4. FTIR Analysis

**Figure 7:**
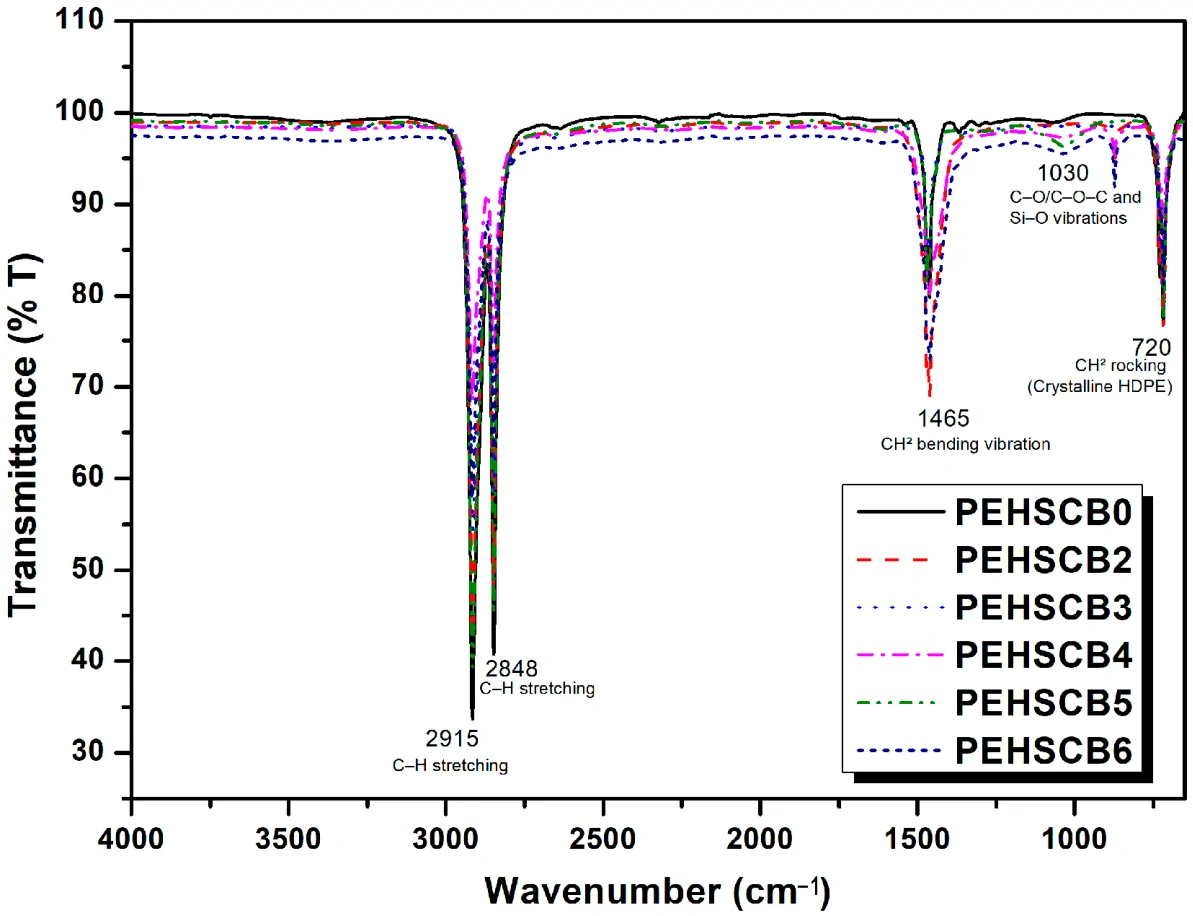
FTIR spectra of neat HDPE (PEHSCB0) and selected composite formulations.

The FTIR spectra of neat HDPE and the selected composite formulations are presented in Figure 7. Neat HDPE (PEHSCB0) exhibited the characteristic absorption bands of polyethylene, with intense bands at approximately 2915 and 2848 cm^−1^, corresponding to the asymmetric and symmetric C-H stretching vibrations, respectively. The bands observed in the region of approximately 1470–1460 cm^−1^ were assigned to CH_2_ bending vibrations, while the characteristic absorption in the 730–720 cm^−1^ region was associated with CH_2_ rocking vibrations of the crystalline polyethylene structure. Comparable characteristic bands have previously been reported for neat HDPE and nutshell-filled HDPE composites [12,13].

The principal polyethylene bands remained dominant in all composite spectra, indicating that the characteristic chemical structure of the HDPE matrix was retained following melt compounding. Nevertheless, formulation-dependent differences were evident in the fingerprint region. In particular, the hazelnut-shell-containing formulations PEHSCB4, PEHSCB5, and PEHSCB6 exhibited absorption bands at approximately 1036, 1031, and 1034 cm^−1^, respectively. Hazelnut shell consists predominantly of lignocellulosic constituents, including cellulose, hemicellulose, and lignin. FTIR analysis of hazelnut shell reported in the literature has revealed O-H stretching at 3402–3340 cm^−1^, C-H stretching at 2924–2855 cm^−1^, C=O stretching at approximately 1711 cm^−1^, aromatic skeletal vibrations in the 1610–1509 cm^−1^ region, and C-O vibrations of carbohydrates and lignin at approximately 1047 cm^−1^ [38]. The bands observed at approximately 1031–1036 cm^−1^ in the present composites can therefore be primarily associated with C-O/C-O-C vibrations originating from the lignocellulosic hazelnut-shell phase. This interpretation is also consistent with previous FTIR observations for nutshell-filled HDPE systems [12,13].

For the talc-containing formulations, an additional contribution from the silicate structure should be considered in this spectral region. Talc exhibits a strong characteristic Si-O stretching vibration at approximately 1018 cm^−1^, together with characteristic Mg_3_O-H and lower-wavenumber vibrations associated with its layered silicate structure [39]. Consequently, the absorption around 1030 cm^−1^ in PEHSCB4 and PEHSCB6 may contain overlapping contributions from C-O/C-O-C vibrations of the hazelnut shell and Si-O vibrations of talc. Importantly, a comparable band was observed at approximately 1031 cm^−1^ in PEHSCB5, which contains hazelnut shell but no talc. This observation supports a substantial contribution of the lignocellulosic component to this region and indicates that the approximately 1030 cm^−1^ band should not be assigned exclusively to talc.

Weak spectral features were also observed at approximately 1600–1620 cm^−1^ in the hazelnut-shell-containing formulations. This region is consistent with aromatic skeletal vibrations associated with lignin, for which hazelnut shell has been reported to exhibit absorption in the approximately 1610–1509 cm^−1^ region [38]. However, because these features were relatively weak and occurred within a multicomponent system, they should be regarded as supportive rather than definitive evidence of the lignocellulosic phase.

No pronounced new absorption bands were evident in the carbonyl region of the composite spectra relative to neat HDPE. This observation is relevant considering the use of maleic-anhydride-modified LLDPE (LLDPE-g-MA) as the compatibilizer. Although maleated polyolefins are commonly employed to enhance interactions between non-polar polyolefin matrices and hydroxyl-containing lignocellulosic fillers, the present FTIR spectra alone do not provide sufficient evidence to establish the formation of new covalent bonds at the polymer–hazelnut-shell interface. Accordingly, possible improvements in interfacial compatibility should be interpreted together with the mechanical and morphological results rather than inferred solely from FTIR analysis.

Overall, the FTIR results demonstrate that the characteristic polyethylene structure was retained after incorporation of the different particulate components. The absorption bands observed at approximately 1031–1036 cm^−1^ in the hazelnut-shell-containing formulations are consistent with C-O/C-O-C vibrations of the lignocellulosic phase, with overlapping Si-O contributions also possible in the talc-containing formulations. The absence of pronounced new absorption bands suggests that the spectral differences predominantly reflect the contributions of the constituent phases rather than major chemical modification of the polyethylene backbone.

### 3.5. Morphological Analysis

**Figure 8:**
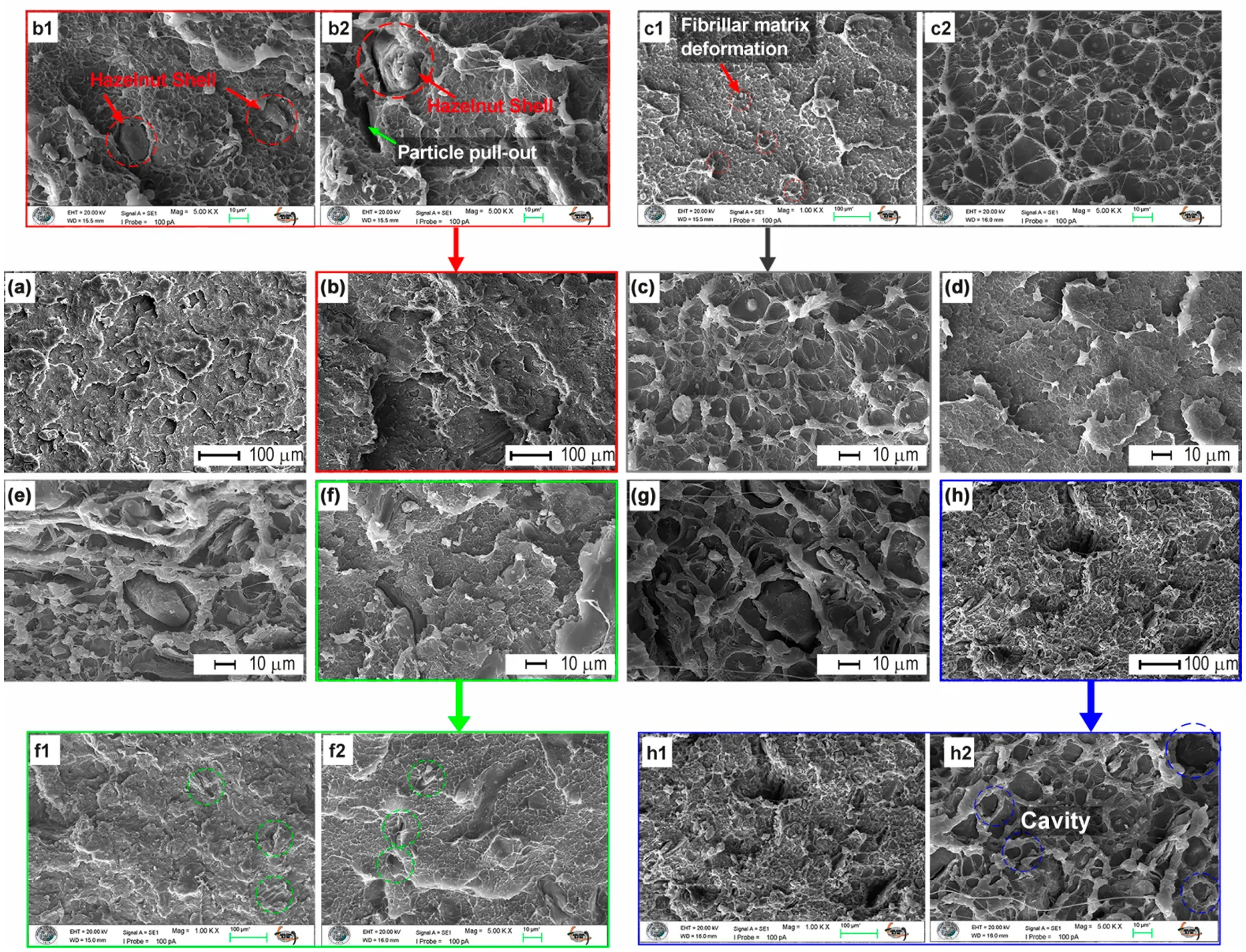
SEM micrographs of the fracture surfaces of (**a**) PEHSCB0, (**b**) PEHSCB1, (**c**) PEHSCB2, (**d**) PEHSCB3, (**e**) PEHSCB4, (**f**) PEHSCB5, (**g**) PEHSCB6, and (**h**) PEHSCB7. Additional micrographs at different magnifications are presented for (**b1**,**b2**) PEHSCB1, (**c1**,**c2**) PEHSCB2, (**f1**,**f2**) PEHSCB5, and (**h1**,**h2**) PEHSCB7. The annotations identify selected morphological features, including hazelnut-shell particles, possible particle pull-out, cavities, and fibrillar matrix deformation.

The fracture-surface morphologies of neat HDPE and the composite formulations are presented in Figure 8. Additional SEM micrographs at different magnifications are included for selected formulations to provide a more detailed examination of particle distribution, interfacial features, and localized fracture deformation. Neat HDPE (Figure 8a) exhibited a comparatively continuous polymer-rich fracture surface characterized by irregular ridges and localized deformation. In contrast, the composite formulations showed increasingly heterogeneous fracture surfaces containing dispersed particulate regions, cavities, irregular fracture paths, and localized matrix deformation. Such features are characteristic of particulate- and natural-filler-reinforced polymer composites, in which the fracture behavior is strongly influenced by filler dispersion and particle–matrix interfacial characteristics [13,40]. In natural-filler/polymer composites, particle or fiber debonding and pull-out may leave cavities on the fracture surface, whereas particles remaining embedded in the matrix can indicate localized mechanical interlocking or interfacial interaction [40]. Accordingly, the morphological features observed here were interpreted together with the mechanical results rather than being used independently as direct evidence of interfacial adhesion.

The individual particulate components produced distinct fracture morphologies. PEHSCB1 (Figure 8b), containing 20 wt.% hazelnut shell, exhibited a considerably rougher and more heterogeneous surface than neat HDPE, with irregular lignocellulosic particles, cavities, and locally deformed polymer regions. These features suggest that fracture involved a combination of matrix deformation, particle fracture, and localized particle debonding or pull-out. The additional micrographs of PEHSCB1 (Figure 8(b1,b2)) provide a closer examination of the irregular hazelnut-shell particles and cavity-like regions within the fractured matrix. Some cavities appear consistent with possible particle pull-out or localized particle–matrix separation during fracture. Nevertheless, these features cannot independently establish the overall quality of interfacial adhesion throughout the composite. Similar heterogeneous fracture behavior has been reported for nutshell-filled HDPE composites, where the distribution of lignocellulosic particles and their interaction with the polyethylene matrix strongly influenced the resulting morphology [13]. PEHSCB2 (Figure 8c) and PEHSCB3 (Figure 8d) also exhibited heterogeneous fracture surfaces, although their morphology differed from that of the hazelnut-shell-containing PEHSCB1. The presence of smaller dispersed features, cavities, and plastically deformed matrix regions in these formulations suggest that the incorporated particulate components influenced local deformation and fracture behavior. The additional PEHSCB2 micrographs (Figure 8(c1,c2)) further reveal fibrillar and locally deformed matrix regions, suggesting that localized plastic deformation contributed to the observed fracture morphology. These morphological observations are consistent with the higher V-notched impact strengths of PEHSCB2 (29.06 kJ/m^2^) and PEHSCB3 (28.88 kJ/m^2^) compared with neat HDPE (12.78 kJ/m^2^). In particular, localized matrix deformation and cavity formation may contribute to energy dissipation during fracture, while crack deflection and particle–matrix debonding represent additional possible mechanisms. However, the observed cavities cannot be unequivocally attributed to particle pull-out or interfacial debonding, and the individual contributions of these mechanisms cannot be quantified from the available SEM images.

More complex fracture morphologies were observed when multiple particulate components were combined. PEHSCB4 (Figure 8e), PEHSCB5 (Figure 8f), and PEHSCB6 (Figure 8g) exhibited irregular particles embedded within the matrix together with cavities, tortuous fracture paths, and pronounced localized matrix deformation. Comparable heterogeneous morphologies have previously been observed in hybrid HDPE composites containing lignocellulosic and inorganic fillers [13]. The morphology of PEHSCB4 is particularly noteworthy when considered together with its mechanical response, as this formulation exhibited the highest flexural strength (38.87 MPa) compared with 35.14 MPa for neat HDPE. Similarly, PEHSCB5, which exhibited the highest tensile modulus (963.3 MPa) and one of the highest Shore D hardness values (71.8), showed a highly heterogeneous fracture surface containing dispersed particulate regions and locally deformed polymer. The additional PEHSCB5 micrographs (Figure 8(f1,f2)) further illustrate this heterogeneous morphology, with localized particle-like features and irregular matrix regions. Although these observations provide qualitative information about the spatial distribution of the dispersed phases, individual particles cannot always be distinguished from possible localized particle clusters based on fracture-surface morphology alone. These observations are consistent with the general principle that the mechanical response of particulate composites depends not only on filler content but also on filler dispersion and the effectiveness of stress transfer across the particle–matrix interface [40]. Nevertheless, the present SEM observations should be regarded as supportive morphological evidence rather than direct proof of improved interfacial adhesion.

The effect of increasing the LLDPE-g-MA content can be considered by comparing PEHSCB6 (Figure 8g) and PEHSCB7 (Figure 8h). Both formulations exhibited rough and heterogeneous fracture surfaces with embedded particles, cavities, and extensive matrix deformation. No unequivocal morphological improvement was evident upon increasing the LLDPE-g-MA content from 3 to 5 wt.%, which is consistent with the absence of a systematic improvement in the mechanical properties of PEHSCB7 relative to PEHSCB6. The additional PEHSCB7 micrographs (Figure 8(h1,h2)) also reveal cavity-like features and localized matrix deformation, indicating that morphological heterogeneity remained evident at the higher compatibilizer concentration. However, these observations do not provide a quantitative measure of interfacial adhesion or demonstrate whether increasing the compatibilizer content improved the dispersion of individual filler components.

Regarding filler agglomeration, the SEM micrographs reveal heterogeneous fracture morphologies with dispersed particle-like features and localized regions of morphological irregularity. Although these observations allow a qualitative assessment of particle distribution, they do not provide sufficient evidence to distinguish unequivocally between individual filler particles, localized particle clustering, and agglomeration, particularly in the formulations containing multiple filler types. Therefore, the absence of agglomeration throughout the composite matrix cannot be conclusively established from the available fracture-surface images. Likewise, the presence of embedded particles and localized cavities should not be interpreted as definitive evidence of strong or weak interfacial adhesion, since fracture morphology may also be influenced by particle geometry, local stress concentration, and deformation during specimen failure.

Overall, the SEM results demonstrate that the transition from neat HDPE to the particulate and hybrid formulations substantially altered the fracture morphology. The mechanical behavior of these materials therefore appears to reflect the combined effects of particle dispersion, local particle–matrix interactions, matrix deformation, and crack propagation through the heterogeneous multiphase structure rather than the effect of any single component alone. This interpretation is consistent with previous observations for nutshell/inorganic-filler HDPE composites and with the established importance of interfacial characteristics in natural-filler/polymer composites [13,40].

### 3.6. Overall Formulation-Property Assessment

The overall results demonstrate that the sequential formulation changes produced distinct property trade-offs rather than a uniform improvement across all measured responses. No single formulation simultaneously maximized stiffness, strength, impact resistance, hardness, thermal behavior, and melt-flow characteristics. This formulation-dependent response is consistent with previous observations for hybrid nutshell/inorganic-filler HDPE composites, where the final performance was governed by the combined effects of composition, filler dispersion, and particle–matrix interactions [13].

Among the investigated formulations, PEHSCB4 provided a favorable mechanical balance, exhibiting the highest flexural strength (38.87 MPa) while maintaining a tensile strength (25.6 MPa) close to that of neat HDPE (26.0 MPa). PEHSCB5 exhibited the highest tensile modulus (963.3 MPa) and Shore D hardness (71.8), whereas PEHSCB2 and PEHSCB3 showed the highest V-notched impact strengths (~29 kJ/m^2^ compared with 12.78 kJ/m^2^ for neat HDPE). The physical, thermal, and morphological analyses further confirmed that these differences were formulation dependent rather than simply proportional to the total particulate content. Moreover, increasing the LLDPE-g-MA content from 3 to 5 wt.% did not provide a systematic overall property improvement. The incorporation of 20 wt.% hazelnut-shell powder partially replaced conventional HDPE resin, reducing its content to 49.17–77 wt.% in the hazelnut-shell-containing formulations. However, these composition-based changes should not be interpreted as equivalent reductions in carbon emissions, which would require a comprehensive life-cycle assessment.

Taken together, these findings indicate that formulation selection should be based on the required balance of properties rather than maximization of a single parameter. For potential rigid food-packaging applications, the present results establish the material-level formulation–property relationships; however, the incorporation of hazelnut-shell powder does not establish suitability for direct food contact. Further application-specific assessments, particularly overall and specific migration testing under the intended conditions of use, are required to evaluate food-contact suitability.

## 4. Conclusions

This study systematically evaluated the formulation-dependent mechanical, thermal, physical, and processing properties of HDPE-based hybrid biocomposites containing hazelnut shell, talc, and carbon black in single-, binary-, and multi-filler combinations using a sequential formulation strategy. The principal contribution of this work lies in the systematic comparison of formulations containing these chemically and morphologically distinct fillers, revealing formulation-dependent property trade-offs within a multi-component HDPE system. In particular, PEHSCB4 exhibited the highest flexural strength (38.87 MPa), while maintaining a tensile strength close to that of neat HDPE. PEHSCB5 provided the highest tensile modulus (963.3 MPa) and Shore D hardness (71.8), whereas PEHSCB2 and PEHSCB3 exhibited the highest V-notched impact strengths, approximately 29 kJ/m^2^ compared with 12.78 kJ/m^2^ for neat HDPE.

The physical, thermal, spectroscopic, and morphological analyses supported this formulation-dependent behavior. All formulations were successfully processed by twin-screw extrusion and injection molding despite substantial differences in melt flow rate. Water absorption increased with hazelnut-shell incorporation, reaching 0.351% for PEHSCB1 after 240 h, whereas the hybrid formulations exhibited slightly lower values (0.317–0.325%). Nevertheless, water absorption remained below 0.36% for all formulations under the conditions investigated. Thermal analysis indicated that the principal degradation region remained well above the processing temperatures used in this study, while FTIR confirmed the presence of the constituent phases without evidence of major chemical modification of the polyethylene backbone. SEM observations revealed heterogeneous multiphase fracture morphologies involving dispersed particles, cavities, irregular fracture paths, and localized matrix deformation. Increasing the LLDPE-g-MA content from 3 to 5 wt.% did not result in a systematic improvement in the overall property profile, suggesting that increased compatibilizer loading was not consistently beneficial.

Overall, the findings demonstrate that the incorporation of hazelnut-shell residue into multi-filler HDPE biocomposites enables composition-dependent tailoring of material properties while maintaining melt processability. The results highlight the importance of balancing mechanical, thermal, and processing characteristics rather than optimizing individual properties independently. The developed materials therefore represent promising candidates for potential rigid food-packaging applications. However, their suitability for direct food contact should be established through further application-specific studies, particularly overall and specific migration testing and, where required, barrier and sensory evaluations.

## Figures and Tables

**Table 1:** Composition of the investigated HDPE-based composites.

Sample Code	HDPE (wt.%)	Hazelnut Shell (wt.%)	Talc (wt. %)	Hostalen CRP 100 BLACK (wt.%)	LLDPE-g-MA (wt.%)
PEHSCB0	100.00	-	-	-	-
PEHSCB1	77.00	20	0	-	3
PEHSCB2	92.00	0	5	-	3
PEHSCB3	74.15	0	0	22.85	3
PEHSCB4	72.00	20	5	-	3
PEHSCB5	55.49	20	0	21.51	3
PEHSCB6	50.60	20	5	21.40	3
PEHSCB7	49.17	20	5	20.83	5

**Table 2:** Tensile and flexural properties of neat HDPE and the developed composite formulations.

Sample Code	Tensile Modulus (MPa)	Tensile Strength (MPa)	Elongation at Break (%)	Flexural Modulus (MPa)	Flexural Strength (MPa)	Flexural Strain at Max. Strength (%)
PEHSCB0	942.9 ± 12.9 ^a^	26.0 ± 0.3 ^a^	271.9 ± 69.2 ^a^	944.9 ± 25.3 ^b^	35.1 ± 0.5 ^bc^	7.47 ± 0.01 ^f^
PEHSCB1	915.2 ± 13.1 ^b^	25.5 ± 0.3 ^ab^	13.7 ± 1.8 ^b^	966.5 ± 28.4 ^ab^	37.0 ± 0.5 ^ab^	8.81 ± 0.16 ^ab^
PEHSCB2	743.5 ± 11.5 ^e^	24.9 ± 0.4 ^bc^	30.6 ± 1.9 ^b^	722.9 ± 11.6 ^c^	31.8 ± 0.5 ^de^	8.23 ± 0.19 ^cd^
PEHSCB3	762.4 ± 12.1 ^e^	23.0 ± 0.3 ^d^	45.2 ± 6.9 ^b^	730.0 ± 7.0 ^c^	30.1 ± 0.4 ^e^	9.00 ± 0.20 ^a^
PEHSCB4	915.0 ± 7.4 ^b^	25.6 ± 0.5 ^ab^	15.1 ± 1.8 ^b^	1018.5 ± 39.3 ^a^	38.9 ± 1.0 ^a^	8.56 ± 0.14 ^bc^
PEHSCB5	963.3 ± 9.9 ^a^	24.6 ± 0.2 ^c^	20.2 ± 2.7 ^b^	958.1 ± 24.0 ^b^	33.2 ± 2.5 ^cd^	8.04 ± 0.16 ^de^
PEHSCB6	875.7 ± 11.0 ^c^	24.7 ± 0.3 ^c^	12.9 ± 1.8 ^b^	928.6 ± 19.4 ^b^	36.0 ± 1.4 ^b^	7.83 ± 0.23 ^e^
PEHSCB7	840.1 ± 6.8 ^d^	24.5 ± 0.2 ^c^	12.8 ± 0.8 ^b^	933.3 ± 40.5 ^b^	37.5 ± 0.9 ^ab^	8.11 ± 0.11 ^de^

Note: Values are presented as mean ± standard deviation (*n* = 5). Different superscript letters within the same column indicate statistically significant differences among formulations (one-way ANOVA followed by Tukey’s HSD test, *p* < 0.05).

**Table 3:** Charpy impact properties of neat HDPE and the developed composite formulations.

Sample Codes	Charpy Impact Properties	
	Unnotched Impact Strength. kJ/m^2^	V-Notched Impact Strength. kJ/m^2^
PEHSCB0	No Break	12.78 ± 0.87 ^e^
PEHSCB1	38.25 ± 21.09 ^b^	15.79 ± 1.15 ^de^
PEHSCB2	147.38 ± 24.46 ^a^	29.06 ± 3.14 ^a^
PEHSCB3	No Break	28.88 ± 1.58 ^a^
PEHSCB4	34.88 ± 1.91 ^b^	18.70 ± 1.04 ^bc^
PEHSCB5	42.94 ± 1.50 ^b^	14.29 ± 0.90 ^de^
PEHSCB6	38.25 ± 1.64 ^b^	17.91 ± 1.52 ^cd^
PEHSCB7	42.30 ± 2.76 ^b^	19.27 ± 0.62 ^bc^

Note: Values are expressed as mean ± standard deviation (*n* = 10). Different superscript letters within the same column indicate statistically significant differences according to one-way ANOVA followed by Tukey’s HSD test (*p* < 0.05). Specimens exhibiting no fracture during the unnotched Charpy test are reported as “No break” and were excluded from the statistical analysis.

**Table 4:** Shore D hardness of neat HDPE and composite formulations.

Sample Code	Shore D Hardness
PEHSCB0	65.8 ± 0.4 ^d^
PEHSCB1	70.0 ± 0.7 ^ab^
PEHSCB2	66.2 ± 0.8 ^cd^
PEHSCB3	71.6 ± 1.1 ^a^
PEHSCB4	68.0 ± 1.6 ^bc^
PEHSCB5	71.8 ± 1.1 ^a^
PEHSCB6	67.0 ± 0.7 ^cd^
PEHSCB7	67.6 ± 1.1 ^cd^

Note: Values are presented as mean ± standard deviation (*n* = 5). Different superscript letters indicate statistically significant differences among formulations according to one-way ANOVA followed by Tukey’s HSD test (*p* < 0.05).

**Table 5:** Comparison of mechanical properties of the developed HDPE biocomposites with literature.

Study	Composite Formulation	Tensile Strength (MPa)	Tensile Modulus (MPa)	Flexural Strength (MPa)	Notched Impact (kJ/m^2^)
Huang and Zhang (2009) [20]	HDPE/20% wood	19.0	830	NR	9.90
Huang and Zhang (2009) [20]	HDPE/50% wood/5 phr compat.	34.4	1750	NR	6.60
Liu et al. (2008) [21]	HDPE/40% bamboo flour	17.5	3640	32.9	2.88
Liu et al. (2008) [21]	HDPE/40% bamboo flour/2.9% PE-g-MA	28.7	3380	49.5	4.58
Karagöz et al. (2025) [13]	HDPE/20% walnut shell	25.6	595.4	46.0	14.48
Karagöz et al. (2025) [13]	HDPE/20% walnut shell/3% nano-CaCO_3_	22.7	596.2	37.3	19.09
Present study	HDPE/20% HS	25.5	915.2	37.0	15.79
Present study	HDPE/5% talc	24.9	743.5	33.2	14.29
Present study	HDPE/20% HS/5% talc	25.6	915.0	38.9	18.70
Present study	HDPE/20% HS/CB	24.6	963.3	33.2	14.29

Note: Impact strength values obtained using different testing methods (Charpy and Izod) are presented for reference and are not directly comparable. NR: Not reported.

**Table 6:** DSC melting and crystallization parameters of neat HDPE and the selected composite formulations.

Sample Code	Δ*H* (J/g)	*Tm* (°C)	Δ*H_C_* (J/g)	*T_C_* (°C)	Xc (%)
PEHSCB0	196.54	138.83	219.95	114.50	67.08
PEHSCB1	157.53	140.83	164.73	111.17	67.21
PEHSCB2	168.04	137.00	188.09	112.33	60.36
PEHSCB4	145.35	137.17	153.88	109.67	66.14
PEHSCB5	178.52	142.83	182.79	118.17	76.60
PEHSCB6	177.59	140.33	188.78	115.67	81.37
PEHSCB7	142.13	135.67	147.81	110.33	65.11

**Table 7:** Thermogravimetric analysis (TGA) parameters of neat HDPE and the composite formulations.

Sample Code	T5 (°C)	T50 (°C)	Tmax (°C)	Residue at 600 °C (%)
PEHSCB0	457.4	482.0	501.0	1.0
PEHSCB1	365.8	481.1	506.1	5.1
PEHSCB2	445.4	484.7	501.8	4.2
PEHSCB3	426.8	488.0	502.1	0.4
PEHSCB4	426.8	485.8	504.7	9.9
PEHSCB5	358.3	485.9	503.9	5.9
PEHSCB6	361.4	486.3	506.3	10.5
PEHSCB7	359.7	486.4	504.2	10.9

**Table 8:** Density and melt flow rate of neat HDPE and the composite formulations.

Sample Code	Density (g/cm^3^)	MFR (g/10 min)
PEHSCB0	0.95 ± 0.01 ^d^	0.22 ± 0.010 ^f^
PEHSCB1	1.01 ± 0.01 ^ab^	1.50 ± 0.100 ^c^
PEHSCB2	0.99 ± 0.01 ^b^	1.00 ± 0.017 ^d^
PEHSCB3	0.95 ± 0.01 ^d^	2.63 ± 0.020 ^b^
PEHSCB4	1.04 ± 0.01 ^a^	0.65 ± 0.010 ^e^
PEHSCB5	1.01 ± 0.01 ^ab^	3.90 ± 0.050 ^a^
PEHSCB6	1.04 ± 0.01 ^a^	0.25 ± 0.010 ^f^
PEHSCB7	1.04 ± 0.02 ^a^	0.25 ± 0.009 ^f^

Note: Values are presented as mean ± standard deviation (*n* = 3). Within each column, values sharing at least one superscript letter are not significantly different according to Tukey’s HSD test (*p* < 0.05), following one-way ANOVA.

## Data Availability

The data presented in this study are available on request from the corresponding authors.
